# Metal Node Control of Brønsted Acidity in Heterobimetallic Titanium–Organic Frameworks

**DOI:** 10.1021/jacs.2c12718

**Published:** 2023-01-23

**Authors:** Ana Rubio-Gaspar, Sergio Navalón, Sergio Tatay, Francisco G. Cirujano, Carmen Fernández-Conde, Natalia M. Padial, Carlos Martí-Gastaldo

**Affiliations:** † Functional Inorganic Materials Team, Instituto de Ciencia Molecular (ICMol), Universitat de València, Paterna, 46980 València, Spain; ‡ Departamento de Química, Universitat Politècnica de València, 46022 València, Spain

## Abstract

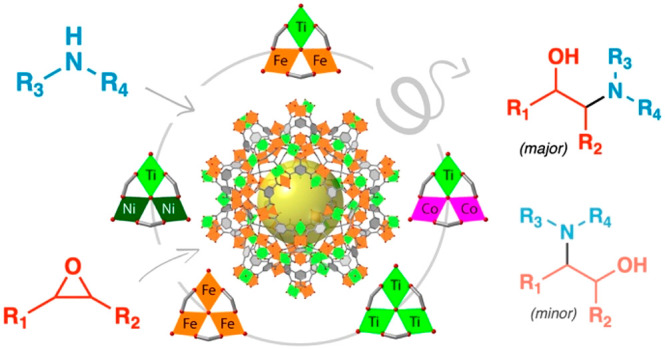

Compared to indirect framework modification, synthetic control of cluster composition can be used to gain direct access to catalytic activities exclusive of specific metal combinations. We demonstrate this concept by testing the aminolysis of epoxides with a family of isostructural mesoporous frameworks featuring five combinations of homometallic and heterobimetallic metal-oxo trimers (Fe_3_, Ti_3_, TiFe_2_, TiCo_2_, and TiNi_2_). Only TiFe_2_ nodes display activities comparable to benchmark catalysts based on grafting of strong acids, which here originate from the combination of Lewis Ti^4+^ and Brønsted Fe^3+^–OH acid sites. The applicability of MUV-101(Fe) to the synthesis of β-amino alcohols is demonstrated with a scope that also includes the gram scale synthesis of propranolol, a natural β-blocker listed as an essential medicine by the World Health Organization, with excellent yield and selectivity.

The vast chemical versatility offered by Metal–Organic Frameworks (MOFs) in terms of structural/topological diversity, sizable porosity, and tailorable pore chemistry is playing an important role in separation, storage, and catalysis technologies.^1^ The precise positioning of organic linkers and inorganic nodes offers unprecedented opportunities for the heterogenization of homogeneous catalysts in a solid matrix for porous crystals with intrinsic catalytic activity.^2^ The design of MOF catalysts has been approached by three main strategies: the use of metal nodes with coordinatively unsaturated sites (open metal sites), the introduction of defects and subsequent generation of coordination vacancies compensated by anionic ligands, or introduction of catalytic units to the organic linker either before framework assembly or postsynthetically. Among them, the first two rely on changes to the coordination geometry or connectivity of the metal nodes in the framework for producing active sites with variable electronic and steric properties for adjustable catalytic activity. This is arguably one of the main characteristics that distinguish MOFs from other synthetic materials, the possibility of using inorganic nodes as tailorable, single-site catalysts.

After the arrival of thermally and chemically stable MOFs, exemplified by MIL-100^3^ and UiO-66^4^ families, this possibility has been exhaustively explored for acid-catalyzed reactions. Generation of unsaturated Lewis acid sites in M_3_ (M = Al^3+^, Cr^3+^, Fe^3+^) and Zr_6_ clusters by thermal treatment or changes to their connectivity can boost their activity in several reactions such as isomerization,^5,6^ condensation,^7^ dehydration,^8^ cycloaddition,^9,10^ or hydrolysis^11,12^ to cite a few. The relative density of Lewis acid sites in the MOF controls its intrinsic catalytic activity, which can be auxiliary improved by treatment with strong inorganic acids such as sulfuric^13^ or sulfonic^14^ acid for immobilization of complementary Brønsted acid sites in the metal cluster or the framework. We argued that Brønsted acidity could be also directly controlled by specific combination of metals in the inorganic node of the framework rather than from indirect modification of an existing cluster. In this way, specific MOF metal sequences would lead to distinctive catalytic activities not reliant on additional modifications.

To prove this concept, here we use the aminolysis of epoxides as a model reaction for testing solid acid catalysis with homometallic MIL-100 and heterobimetallic titanium MUV-101 MOFs. Both families of MOFs are isostructural and based on equivalent [M_3_(μ_3_-O)(O_2_CR)_6_] (M = Fe^3+^, Ti^4+^, Co^2+^, Ni^2+^; R = trimesic acid) clusters for systematic comparison of variable metal-oxo M_3_ trimer combinations: Fe_3_, Ti_3_, TiFe_2_, TiCo_2_, and TiNi_2_ (Figure 1). Our results reveal that TiFe_2_ nodes display intrinsic catalytic activities for the amination of epoxides that are comparable to benchmark MOF catalysts modified with strong acids and originate from the combination of open metal Lewis Ti^4+^ and Brønsted Fe^3+^–OH sites only present in this framework.

**Figure 1:**
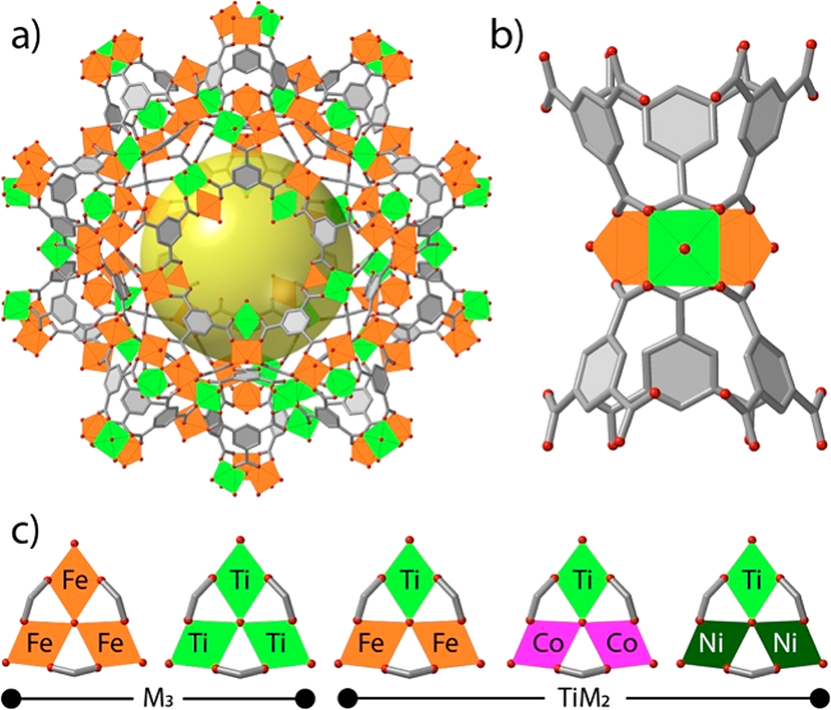
(a) Structure of titanium MUV-101 frameworks built from the interlinking of (b) heterobimetallic TiM_2_ trimers. (c) Mono- and bimetallic metal node combinations in MIL-100 and MUV-101.

MUV-101 and MIL-100 materials were synthesized according to the reported protocols.^15−17^ Phase purity was confirmed by powder X-ray diffraction (PXRD), scanning electron microscopy (SEM), and N_2_ adsorption. Energy dispersive X-ray spectroscopy (EDX) single-point mapping measurements were used to confirm metal ratios in the bimetallic nodes of the MUV-101 family (Supplementary Section S2). The presence of heterobimetallic clusters in this family of materials was previously demonstrated with EXAFS^15^ and PDF^16^ analysis. We used in situ FT-IR spectroscopic studies of carbon monoxide adsorption to investigate the nature and relative densities of acid sites in all solids. The strong dipole moment of CO, and the sensitivity of its stretching frequency to the acidity of the site it is bound to, make it an ideal probe for characterizing changes to the electronic nature of exposed metal sites. Polycrystalline solids were pressed into self-supported wafers and evacuated at 423 K (10^–6^ mbar) prior to CO dosing at 118 K (Figure 2a). Figure 2b shows the FT-IR spectra of all MOFs at low and high CO adsorption pressures normalized to the maximum intensity measured in the 2200–2150 cm^–1^ range. In general, all the spectra at low CO pressure are dominated by the binding of CO to Lewis acid sites (2200–2150 cm^–1^); the sharper features visible for the MUV-101 family support higher crystallinity compared to their homometallic MIL-100 counterparts, which is also consistent with their PXRD patterns. The presence of onset CO features centered at 2189 cm^–1^ suggests small changes in the acidity of uncoordinated metal sites. FT-IR spectra of CO adsorbed at saturation pressure show the appearance of a band at 2135 cm^–1^ associated with CO physisorption and exhibit a shift of the Lewis band maxima to lower frequency values with respect to CO adsorption at low pressures, which can be attributed to the progressive coverage of weaker acid sites and/or adsorbate–adsorbate interactions. Remarkable is the observation of a slight but significant shift toward higher wavenumbers of Lewis acid site maximum of MUV-101(Fe) at 2176 cm^–1^ compared to that of MIL-100(Fe) at 2174 cm^–1^. This increase in the Lewis acidity can be associated with the presence of Ti^4+^ in MUV-101(Fe). In addition, some samples exhibit a band centered at 2154 cm^–1^ associated with Brønsted sites hydrogen bonded to CO molecules.^17^ Along the heterometallic series, this contribution is only present in MUV-101(Fe) arguably due to the formation of a H-bond between CO and the M–OH sites that are exclusive to the local structure reported for this cluster (Figure 2c).^16^ The presence of such interactions is confirmed by the gradual decrease of the −OH vibration band at 3662 cm^–1^ upon CO adsorption with the concomitant appearance of new bands at about 3600 and 3535 cm^–1^ (Figure S6).

**Figure 2:**
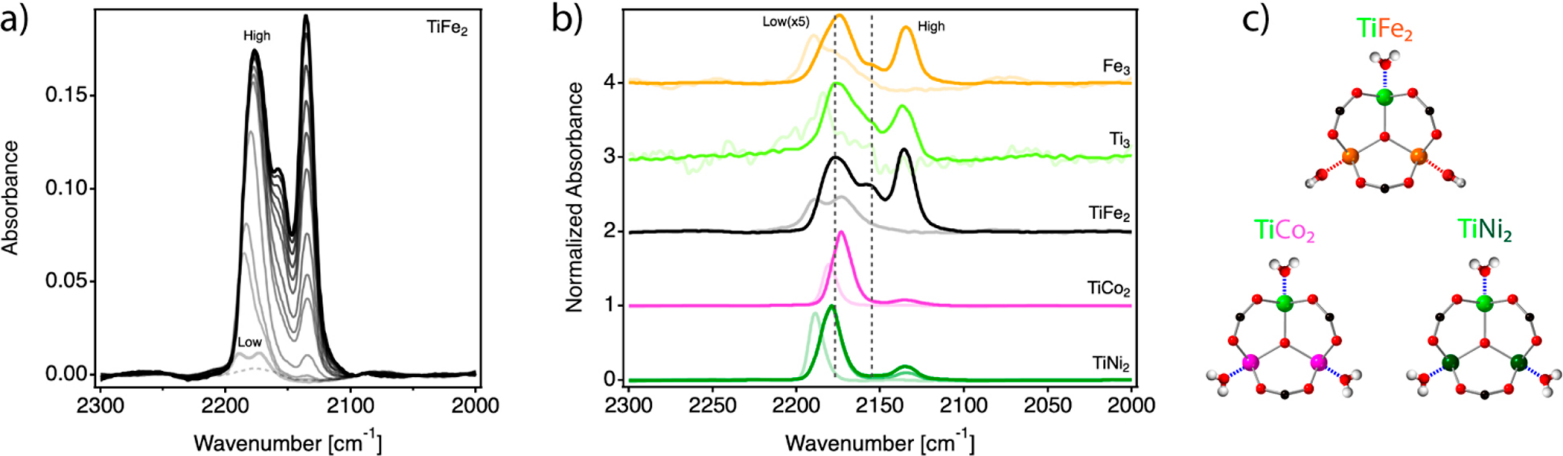
(a) FT-IR spectra at 118 K of MUV-101(Fe) after the introduction of increasing doses of CO. Dotted line, before CO adsorption; thick gray line, CO pressure of 0.08 mbar (low coverage); solid thick black line, after the introduction into the cell of an equilibrium pressure of 288 mbar (high coverage). (b) Normalized IR spectra of MUV-101(M), MIL-100(Fe), and MIL-100(Ti) at high and low CO adsorption pressures. (c) Local structure of heterobimetallic TiM_2_ trimers in the MUV-101 family. The excess of charge due to the introduction of Fe^3+^ metals is counterbalanced by the coordination of OH^–^ anions only in MUV-101(Fe).

We used the epoxide ring-opening amination reaction as a model catalytic test for discriminating the Brønsted acidity intrinsic to TiFe_2_ clusters.^18^ We tested the activity of the materials under study for catalyzing the nucleophilic addition of aniline (**2**) to cyclohexene oxide (**1**) to yield 2-(phenylamino)cyclohexan-1-ol (**3**). In all cases, full selectivity toward compound **3** was observed. As shown in Figure 3a, only MUV-101(Fe) yields complete conversion at 30 °C (2% mol catalyst). Neither MIL-100 phases, nor the rest of heterometallic MUV-101 solids reach conversions above 60%, and all suffer from much slower reaction kinetics (Figure 3b). This is also the case for a physical mixture of MIL-100(Fe) and MIL-100(Ti) in a 2:1 ratio, which suggests that the activity of MUV-101(Fe) cannot be reproduced by using equivalent chemical compositions and shall be instead dictated by the presence of TiFe_2_ heterobimetallic clusters. The compositional analysis of all heterometallic MOFs confirm cluster connectivity indexes near to ideal, thus discarding defectivity as the origin of changes in the catalytic activity (Figure S2). The heterogeneous nature of the reaction was confirmed with hot-filtration tests and ICP analysis that were used to discard metal leaching from the solid to the solution during the reaction (Supplementary Section S.7.6). In line with our FT-IR spectroscopic studies of adsorption of CO, the interplay between higher Lewis acidity and the presence of Brønsted acid sites specific to this cluster seems to be responsible for its distinctive activity. To confirm this point, we tested the catalytic activity of MUV-101(Fe) in the presence of 2,6-lutidine and pyridine, for selective blocking of Brønsted or Lewis sites respectively (Figure 3c).^19,20^ Our results confirm a drop in the activity after the poisoning of the catalyst in both cases, suggesting that an interplay between both acidities is required for reaching complete conversion. Compared to pyridine, the effect of lutidine is more drastic, thus supporting the dominant role of Brønsted acidity in our case. Compared to the iron or titanium Lewis acid activation of the epoxide, which is operative in all clusters, only the Brønsted acidity of the TiFe_2_ nodes in MUV-101(Fe) would enable a dual activation mechanism in which the additional protonation of the epoxide would make it more electrophilic and a better leaving group to facilitate the nucleophilic attack by the amine. The proton transfer ability of this cluster is also responsible for the distinctive performance of this MOF for the hydrolysis of P–F bonds in non-buffered conditions, which according to acid–base titration experiments was associated with the higher density of hydroxyl sites intrinsic to MUV-101(Fe).^16^

**Figure 3:**
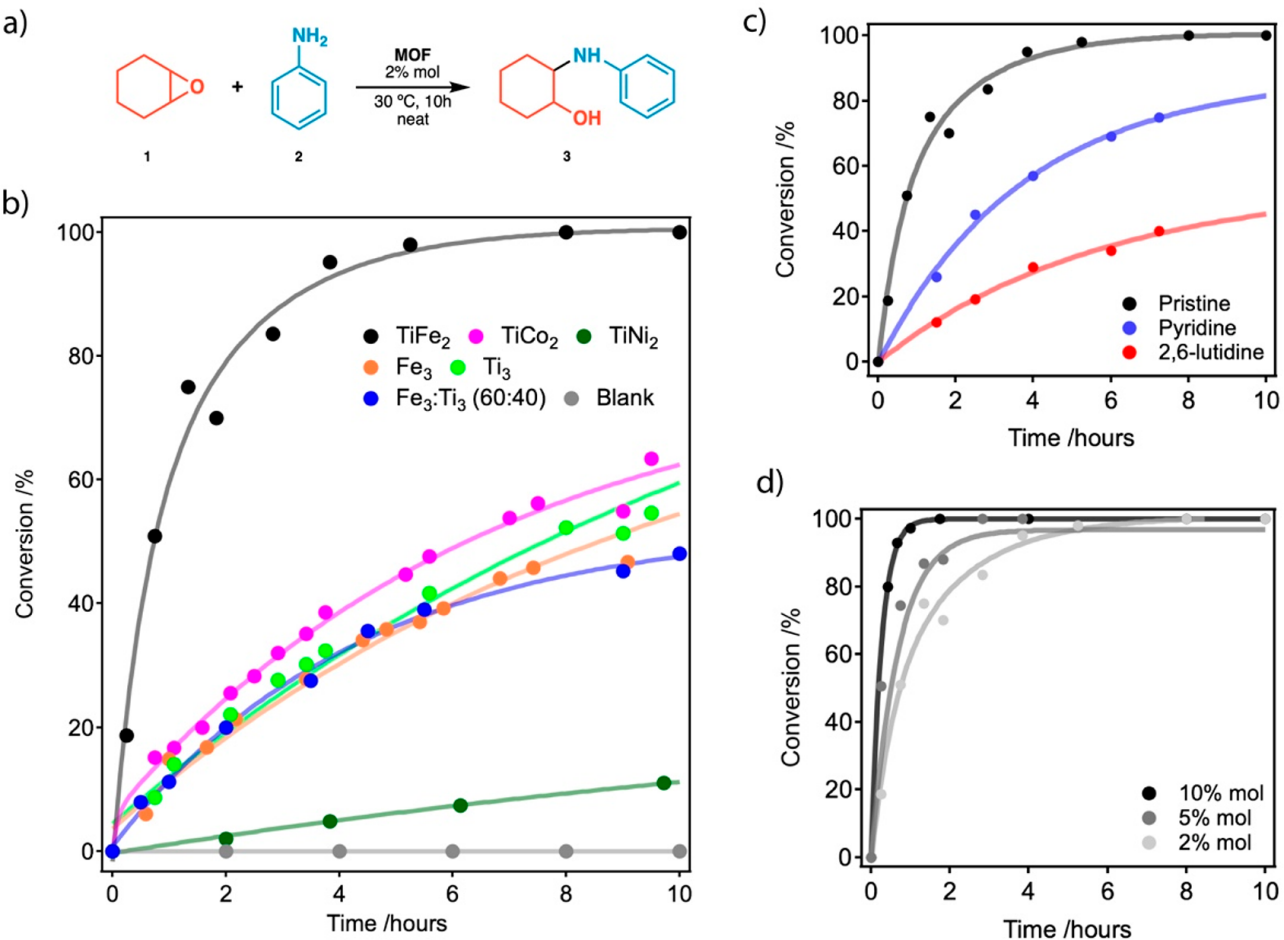
(a) Epoxide ring-opening amination model reaction used as for discriminating Brønsted acidity. (b) Catalytic profiles of heterometallic MUV-101, homometallic MIL-100, and a physical mixture of iron and titanium MIL-100 phases in a 2:1 molar ratio. (c) Influence of the presence of pyridine and 2,6-lutidine on the catalytic activity of MUV-101(Fe). (d) Influence of the MUV-101(Fe) amount on the reaction kinetics. Supplementary Section S4 contains all details relevant to the methodology used. Solid lines are only a guide to the eye.

We then sought to check the influence of the catalyst amount over the reaction kinetics (Figure 3d). MUV-101(Fe) reaches full conversion and selectivity for all concentrations tested with an increase in the initial reaction rate (*k*) of 3.6 h^–1^ and a half-life (*t*_1/2_) of 0.19 h for a 10% mol catalyst loading. The conversion plot for 2 and 5% mol of catalyst would correspond to TOF and TON numbers of 39 h^–1^ and 78 (2 h). Also important, MUV-101(Fe) displays excellent recyclability with unchanged performance and full conversion after four cycles that confirms the durability and lifetime of the catalyst (Supplementary Section S.7.5). This is consistent with the negligible changes to the crystallinity, particle morphology, or accessible porosity of the catalyst confirmed by PXRD Le Bail refinement, SEM measurements, and gas adsorption analysis (Supplementary Section S7). Chemical decomposition or structural collapse of the other MIL-100 and MUV-101 materials under these conditions were also discarded with PXRD and ICP analysis to confirm the direct effect of metal node composition over activity.

The activity of MUV-101(Fe) for this epoxide-ring opening amination surpasses most MOFs reported in this context (Table S3) and meets that of the benchmark material ZrOTf-BTC.^20^ However, whereas the activity of ZrOTf-BTC originates from the treatment of MOF-808 with hydrochloric acid and trimethylsilyl triflate (Me_3_SiOTf) for replicating the strong Lewis acidity of the homogeneous catalysts Sc(OTf)_3_, the activity of MUV-101(Fe) is intrinsic to its framework, does not require auxiliary modifications, and is specific to the heterobimetallic TiFe_2_ cluster. These observations encouraged us to generalize the value of MUV-101(Fe) as a general catalyst for acid-catalyzed nucleophilic ring-opening aminations. This is a classical reaction for the synthesis of β-amino alcohols, which are important building blocks for the synthesis of biologically and pharmacologically active molecules often restricted by the low nucleophilicity of aromatic or sterically hindered amines that impose low yields or aggressive conditions. At 30 °C and 5% mol loadings, MUV-101(Fe) displays excellent activity for the reaction of aniline (**2**) with several epoxides including halogenated, alkylic, or aromatic 2-oxiranes and cyclopentene oxide to form the corresponding amino alcohols in yields between 99 and 80% (Table 1). We observe excellent to good regioselectivities of 95–60% for the aminolysis of unsymmetrical epoxides. As expected, the reaction always favors the formation of the alcohols with Markovnikov regioselectivity as results from the attack on the less substituted carbon of the unsymmetrical epoxide.^21^ We also tested the reactivity of cyclohexene oxide (**1**) with several aromatic amines including variable degrees of electronic activation and steric hindrance in the same conditions. The introduction of −F, −Br, or −Cl substituents in different positions of the aromatic ring for electron-deficient derivatives also proceeds with high yields, above 90% except for 4-bromoaniline (**29**) to give the corresponding β-amino alcohol **13**. The use of electron-rich primary (**32**) or secondary anilines (**31**) with steric hindrance decreases the activity down to 75% isolated yields of the corresponding products (compounds **15** and **16**).

**Table 1:**
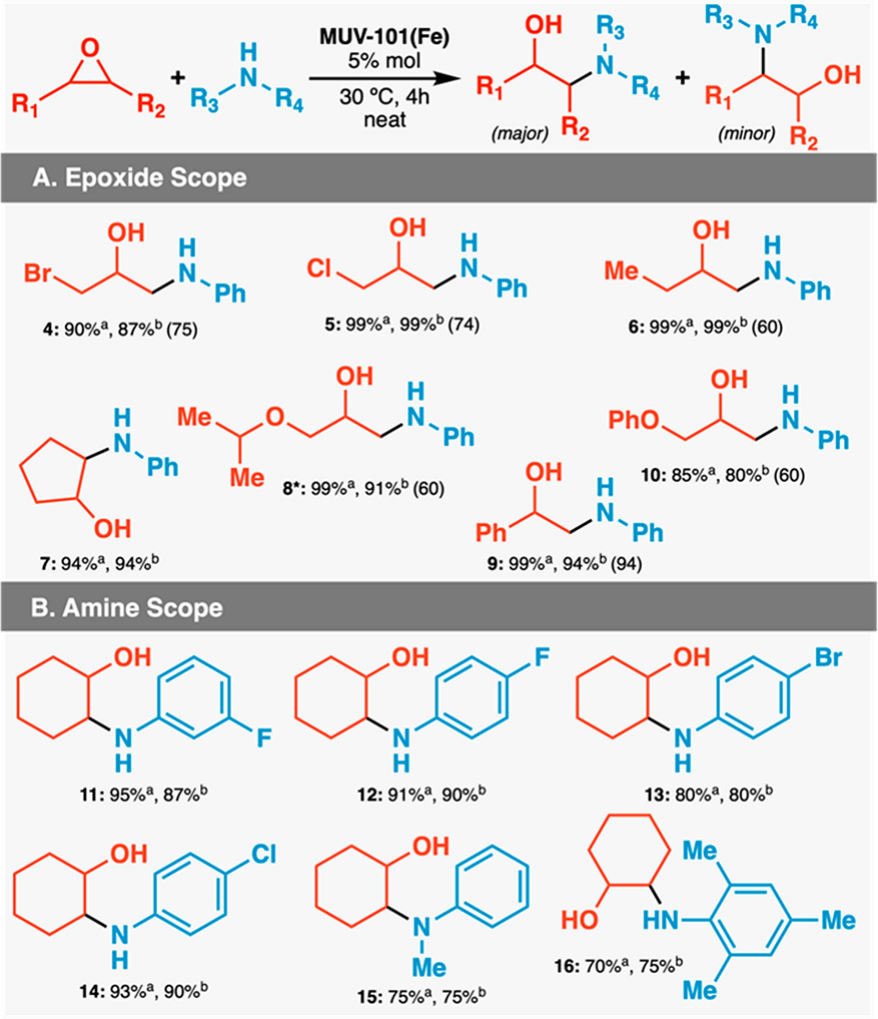
Synthesis of β-Amino Alcohols with MUV-101(Fe)^c^

a Yield determined by GC-FID with dodecane as an internal standard.

b Isolated yield. Yield of the major regioisomer is shown in parentheses.

c Reactions were carried out on a 0.32 mmol scale by using 1.0 equiv of epoxide and 1.0 equiv of amine. *Reaction time: 2 h.

These results pushed us to go one step forward and attempt the synthesis of propranolol (**19**), taking advantage also of the mesoporosity of the framework. Propranolol is a natural β-blocker used to treat high blood pressure and other cardiovascular conditions listed as an essential medicine by the World Health Organization. It is often prepared by homogeneous catalysis, and the examples of heterogeneous alternatives are scarce and limited to strongly acidic supports.^22^ To the best of our knowledge, there are no precedents for the synthesis of this compound by using MOFs as a catalyst. Reaction of naphthyl glycidyl ether (**17**) with propan-2-amine (**18**) at 50 °C and loadings of 5% mol of MUV-101(Fe) give excellent isolated yields and regioselectivity for the formation of propranolol (**19**) also at gram scale (Scheme 1).

**Scheme 1:**
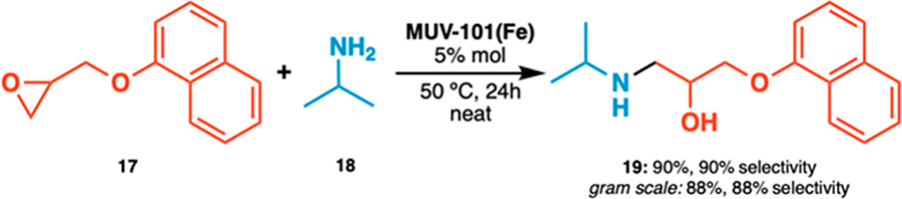
Synthesis of Propranolol Reaction was carried out on a 0.32 mmol scale by using 1.0 equiv of epoxide (**17**) and 1.0 equiv of amine (**18**).

Our results reveal how the control over cluster composition in heterobimetallic frameworks can be used to access catalytic activities that are exclusive to specific metal combinations. Together with our recent works on dual metal catalysis^16^ or the synthesis of mixed oxides with unprecedented stoichiometries,^23^ these results underpin the potential of programming metal combinations for targeted functions. Combined with the outstanding chemical stability and photoredox properties of titanium MOFs, we are confident this same concept can be extended to other multimetallic frameworks for tailorable functions derived from the synergistic interaction of metal centers.

## Supporting Information Available

Synthetic and experimental details, physical characterization, supporting tables and figures (PDF)
