# Supplementary material for: Metal Node Control of Brønsted Acidity in Heterobimetallic Titanium–Organic Frameworks
Source: J Am Chem Soc. 2023 Jan 23;145(7):3855–60. doi: 10.1021/jacs.2c12718 (PMC9951219; doi:10.1021/jacs.2c12718)
Supplement: Supplementary file 1 — ja2c12718_si_001.pdf [file ja2c12718_si_001.pdf]

# Supporting Information

## Metal Node Control of Brønsted Acidity in Heterobimetallic Titanium-Organic Frameworks

Ana Rubio-Gaspar, Sergio Navalón, Sergio Tatay, Francisco G. Cirujano, Carmen Fernández-Conde, Natalia M. Padial, and Carlos Martí-Gastaldo

**Table of Contents**

**S.1 GENERAL CONSIDERATIONS: STARTING MATERIALS AND CHARACTERIZATION ............S3**

S.1.1 Materials and reagents ........................................................................................S3

S.1.2 Physical and chemical characterization ................................................................S3

**S.2 SYNTHESIS AND CHARACTERIZATION OF MATERIALS ........................................................S4**

**S.3 FTIR-CO ADSORPTION MEASUREMENTS ..........................................................................S8**

**S.4 CATALYTIC STUDIES ...........................................................................................................S9**

S.4.1 Catalytic Procedure ...............................................................................................S9

S.4.2 Yield calculation with GC-FID.................................................................................S9

S.4.3 First order kinetic fitting for cyclohexene oxide opening with aniline using MUV-101(Fe) as catalyst.................................................................................................................S10

**S.5 POISONING OF THE CATALYST ..........................................................................................S11**

**S.6 COMPARISON WITH REPRESENTATIVE MATERIALS ..........................................................S12**

**S.7 STABILITY AND RECYCLABILITY OF MUV-101(M) AND MIL-100(M) AS CATALYST ...........S13**

S.7.1 PXRD of MUV-101(M) after catalysis .....................................................................S13

S.7.2 Le Bail Refinement of used MUV-101(Fe) ..............................................................S14

S.7.3 SEM images of the used materials.........................................................................S15

S.7.4 $N_2$ Adsorption of MUV-101(Fe) after catalysis.......................................................S16

S.7.5 Recyclability tests for MUV-101(Fe) .......................................................................S17

S.7.6 ICP and Hot-Filtration Test .....................................................................................S17

**S.8. SCOPE OF THE REACTION ..................................................................................................S19**

**S.9 REFERENCES .........................................................................................................................S36**

## S.1 GENERAL CONSIDERATIONS: STARTING MATERIALS AND CHARACTERIZATION

### S.1.1 Materials and reagents

$Ti(O^iPr)_4$, $FeCl_2·4H_2O$, $CoCl_2·6H_2O$, $NiCl_2·6H_2O$, $MgCl_2·6H_2O$, *N,N*-dimethylformamide, acetic acid, cyclohexene oxide, 2.6-lutidine, pyridine, epichlorohydrin, epibromohydrin, 1,2-epoxybutane, cyclopentene oxide, Isopropyl glycidyl ether, phenyl glycidyl ether, styrene oxide, 3-fluoroaniline, 4-fluoroaniline, 4-bromoaniline, 4-chloroaniline, *N*-methylaniline and 2,4,6-trimethylaniline were purchased from Merck. Benzene-1,3,5-tricarboxylic acid and aniline were purchased from TCI Europe. *n*-Dodecane was purchased from ABCR. Naphthyl glycidyl ether was purchased from Biosynth Carbonsynth. All reagents and solvents were used as received without further purification.

### S.1.2 Physical and chemical characterization

- **X-Ray Diffraction (XRD)** patterns were collected in a PANalytical X'Pert PRO diffractometer using copper radiation (Cu Kα = 1.5418 Å) with an X'Celerator detector, operating at 40 mA and 45 kV. Profiles were collected in the 2° < 2θ < 40° range with a step size of 0.017°.

- **Scanning Electron Microscopy (SEM)** and single point energy-dispersive X-Ray analysis (EDX): particle morphologies, dimensions and mapping were studied with a Hitachi S-4800 scanning electron microscope at an accelerating voltage of 20 kV, over metalized samples with a mixture of gold and palladium during 90 seconds.

- **Gas Adsorption** measurements were recorded on a Micromeritics 3Flex apparatus at relative pressures up to 1 atm. The sample was degassed overnight at 60 °C and $10^{-6}$ Torr prior to analysis. Surface area, pore size and volume values were calculated from $N_2$ adsorption-desorption isotherms (77 K) Specific surface area was calculated by multi-point Brunauer-Emmett-Teller (BET) method. Total pore volume values were taken at $P/P_0$=0.96. Pore size distributions were analysed by using the solid density functional theory (NLDFT) for the adsorption branch by assuming a cylindrical pore model.

-**Fourier-Transform Infrared CO Adsorption** experiments were carried out in a Nexus 8700 FTIR spectrophotometer equipped with an infrared cell that allows in situ treatments at controlled temperature and connected to a high vacuum system with gas dosing facility.

-**Gas Chromatography with Flame Ionisation Detector (GC-FID)** was used to examine the course of the reaction. The equipment was an Agilent 6890N with a column installed of the following characteristics: Agilent 199091J-413 HP-5 with the following dimensions: 30 m x 320 µm x 0.25 µm.

- **Inductively Coupled Plasma Mass Spectroscopy (ICP-MS)** was performed by digestion in a microwave oven. Analysis was performed in an Agilent 7900 ICP-MS.

- **Nuclear Magnetic Resonance (NMR)** spectra were recorded on a Bruker Avance III 300 WB spectrometer and were calibrated to the residual solvent peak ($CDCl_3$ at 7.26 ppm for $^1$H-NMR and 77.36 ppm for$^{13}$C-NMR). The following abbreviations were used to explain multiplicities: s = singlet, d = doublet, t = triplet, q = quartet, m = multiplet.

**- High Resolution Mass Spectrometry (HRMS)**: High resolution quadrupole time-of-flight mass spectrometer, QTOF (LC-MS/MS). The experiments were carried out with TripleTOF™ 5600 LC/MS/MS System, (AB SCIEX) apparatus. Ionization source: ESI/APCI. Temperature: 450 ºC, ion spray voltage (ISVF): 5500.

## S.2 SYNTHESIS AND CHARACTERIZATION OF MATERIALS

Materials were synthesised following the reported procedures for MIL-100(Ti),[1] MIL-100(Fe)[2] and MUV-101(M).[3] The corresponding analyses (XRPD, SEM, EDX, and $N_2$ adsorption) were carried out to confirm the successful synthesis of the materials.

### X- Ray Powder Diffraction (PXRD)

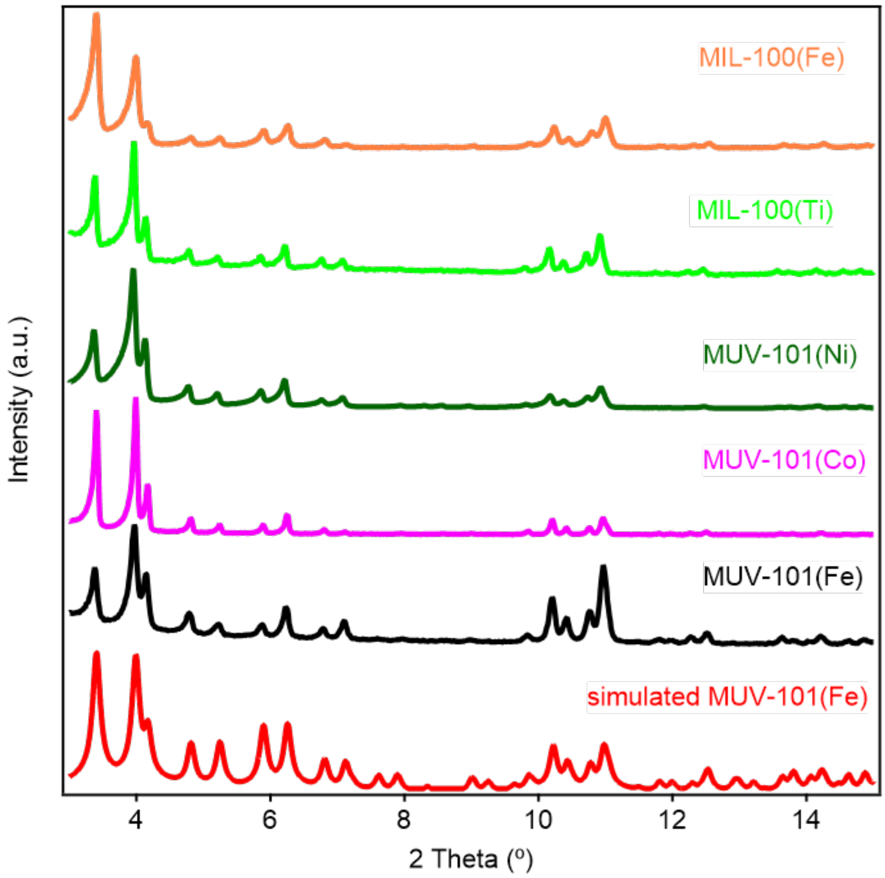


**Figure S1.** From top to bottom: X-Ray diffraction patterns of MIL-100(Fe), MIL-100(Ti), MUV-101(Ni), MUV-101(Co), MUV-101(Fe) and simulated pattern of MUV-101(Fe) calculated from the structural file CCDC 1960226.[1]

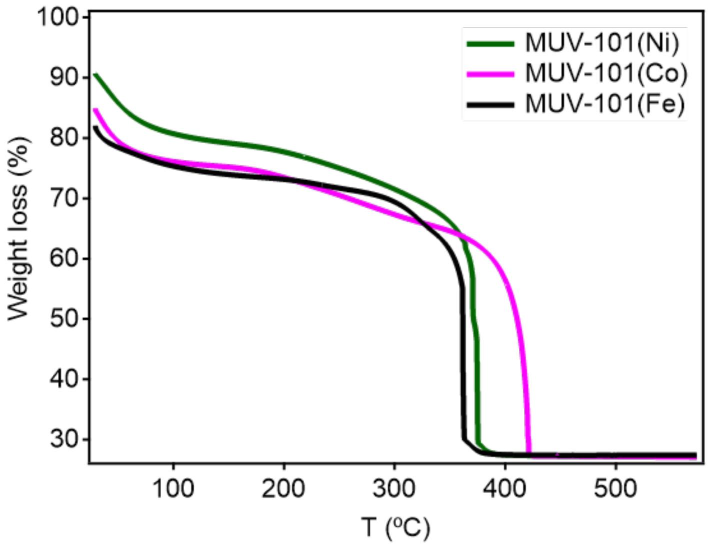


**Figure S2.** Thermogravimetric analysis of MUV-101(Ni), MUV-101(Fe) and MUV-101(Co).

The weight loss between 300 and 450 ºC was used to calculate the number of btc linkers per cluster by assuming that: i) any missing btc linker would be compensated by the inclusion of two acetate molecules, ii) acetate molecules would decompose before btc, and iii) after acetate decomposition charge would be compensated by oxide anions. This would agree with the formula: $Cluster_1\ btc_{2-x}\ O_{3/2x}$

Compared to the theoretical cluster connectivity index of 6, we observe minimum deviations between 5-6 for all sample that rule out significant defectivity changes in these heterometallic frameworks.

**Scanning Electron Microscopy (SEM)**

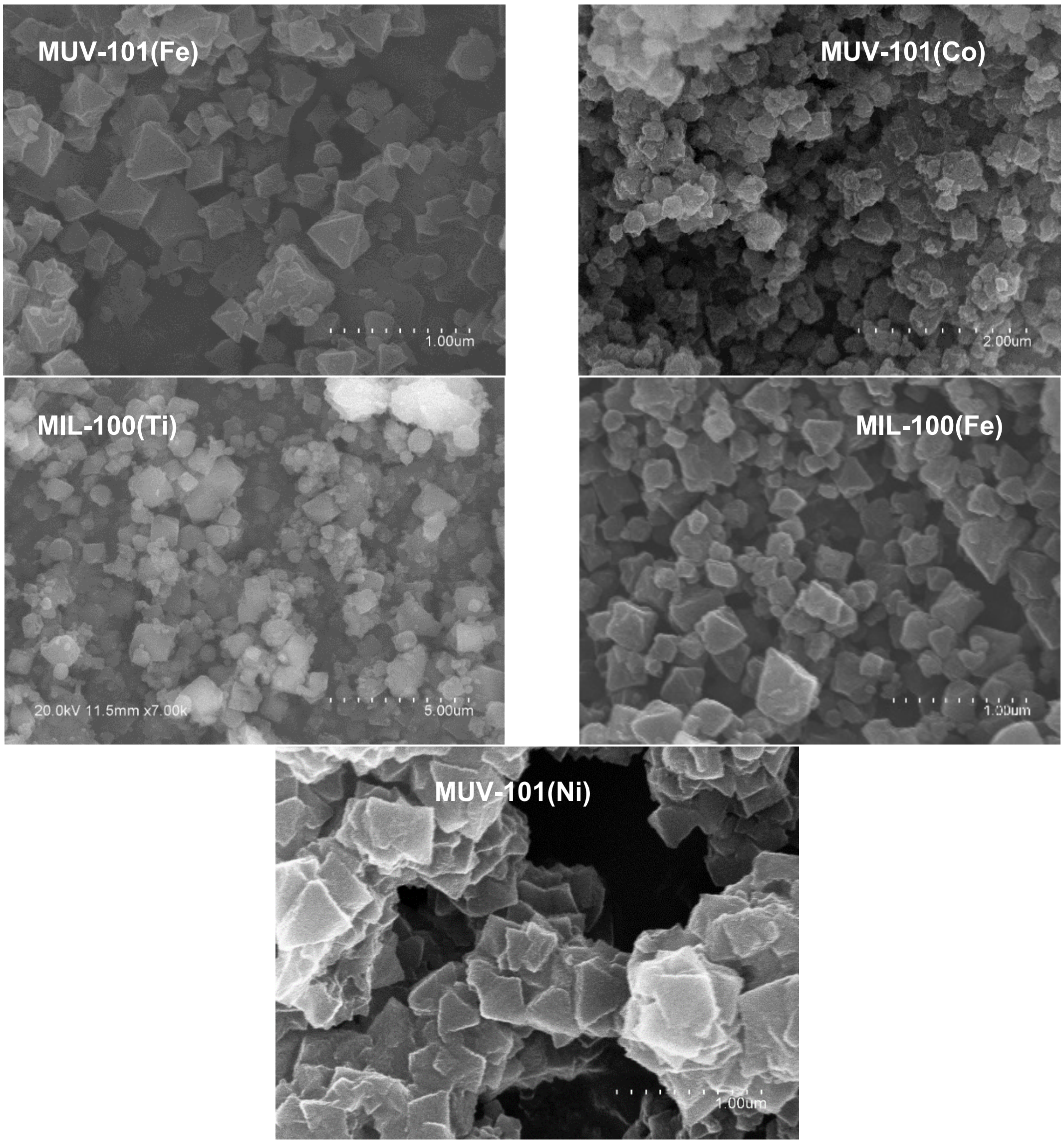


**Figure S3.** Scanning Electron Microscopy images of the materials used in this work.

## Energy-dispersive X-Ray analysis (EDX)

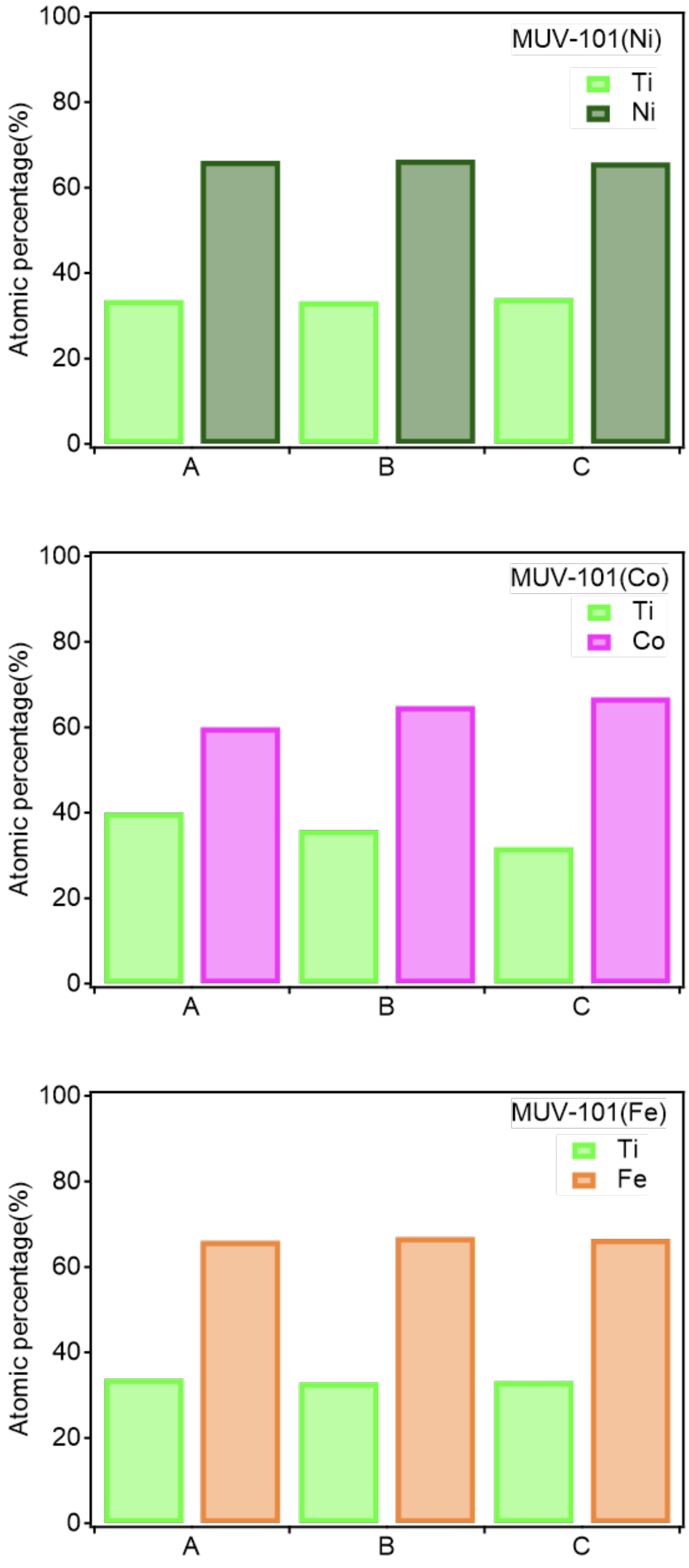


**Figure S4.** Experimental Ti:M ratio from point and shoot EDX analysis for different crystals.

## Inductively Coupled Plasma Mass Spectroscopy (ICP-MS)

**Table S1.** Total metallic content determined by ICP-MS.

| Material | Ti (%) | Fe (%) | Co (%) | Ni (%) |
|---|---|---|---|---|
| MUV-101(Co) | 49 | | 51 | |
| MUV-101(Fe) | 35 | 65 | | |
| MUV-101(Ni) | 39 | | | 61 |

## Nitrogen adsorption isotherms

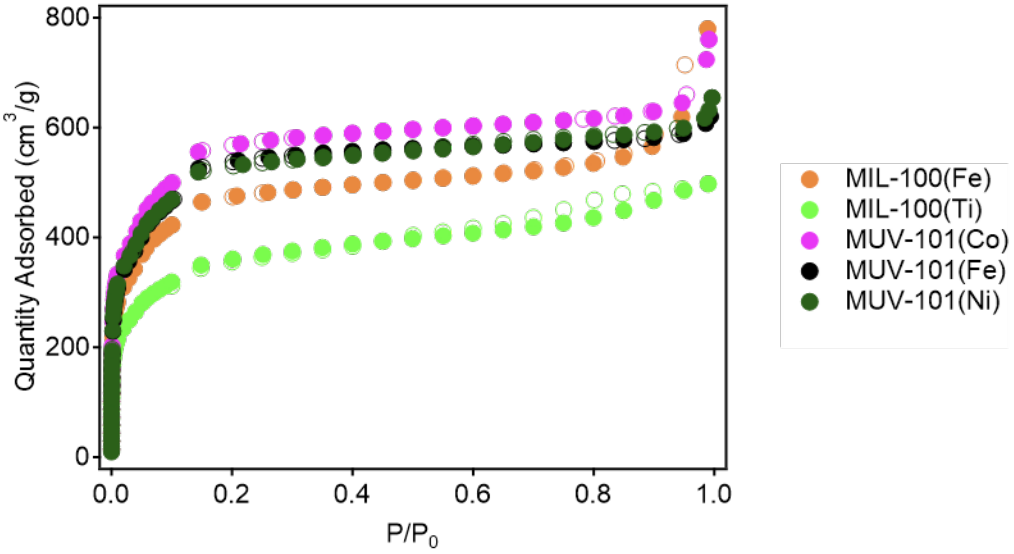


**Figure S5.** $N_2$ adsorption isotherms of the materials used in this work.

**Table S2.** Experimental BET surface area of the materials used in this work.

| Material | BET value ($m^2/g$) |
|---|---|
| MIL-100(Fe) | 1809.8 ± 9.3 |
| MIL-100(Ti) | 1346 ± 15.2 |
| MUV-101(Co) | 2138.6 ± 11.1 |
| MUV-101(Fe) | 1972.7± 20.0 |
| MUV-101(Ni) | 1985 ± 1.2 |

### S.3 FTIR-CO ADSORPTION MEASUREMENTS

Approximately 6 mg of the polycrystalline solids were pressed into self-supported wafers and treated under vacuum (10-6 mbar) at 423 K for 5 h. Subsequently, the wafers were cooled down to 118 K under dynamic vacuum followed by CO dosing at increasing pressure. After each CO dosage the FT-IR spectrum was recorded until MOF saturation. Data was baseline corrected using a poly 5 function.

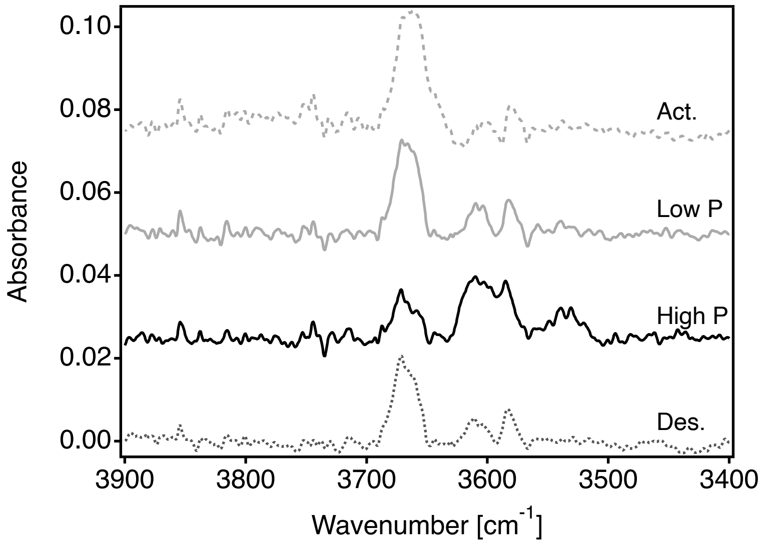


**Figure S6.** FT-IR spectra at 118 K of MUV-101(Fe) after activation at 423 K for 4h (Act.), the introduction of 0.08 mbar of CO (Low P), the introduction of an equilibrium pressure of 288 mbar (High P) and CO desorption through chamber evacuation at $10^{-6}$ mbar (Des.).

## S.4 CATALYTIC STUDIES

### S.4.1 Catalytic Procedure

Prior to the reaction, the material is activated overnight in a vacuum oven at 150ºC.

General procedure:

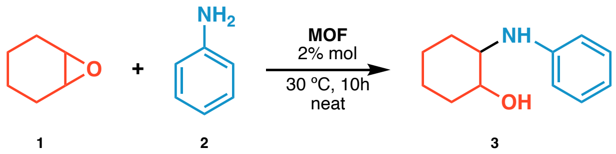


1 equivalent of oxirane (**1**), 1 equivalent of amine (**2**) and 80 μL (0.35 mmol) of *n*-Dodecane as internal standard were added to a 2 mL vial containing 2%, 5% or 10% of the corresponding activated MOF [(MUV-101(M) M= Fe, Co, Ni or MIL-100 (Fe or Ti)]. The mixture was stirring during 10h at 30ºC. Yields determined by GC-FID.

Different catalyst loading experiments:

**2% loading**: Following the general procedure 0.80 mmol of aniline (75 μL), 0.80 mmol of cyclohexene oxide (80 μL) and 0.35 mmol of *n*-Dodecane (80 μL) as internal standard were added to a 2 mL vial containing 10 mg of the corresponding activated MOF [(MUV-101(M) M= Fe, Co, Ni or MIL-100 (Fe or Ti)]. The mixture was left stirring during 10h at 30ºC. Yields determined by GC-FID.

**5% loading**: Following the general procedure 0.32 mmol of aniline (30 μL), 0.32 mmol of cyclohexene oxide (32 μL) and 0.35 mmol of *n*-Dodecane (80 μL) as internal standard were added to a 2 mL vial containing 10 mg of MUV-101(Fe). The mixture was left stirring during 10h at 30ºC. Yield determined by GC-FID.

**10% loading**: Following the general procedure 0.16 mmol of aniline (15 μL), 0.16 mmol of cyclohexene oxide (16 μL) and 0.35 mmol of *n*-Dodecane (80 μL) as internal standard were added to a 2 mL vial containing 10 mg of MUV-101(Fe). The mixture was stirring during 10h at 30ºC. Yield determined by GC-FID.

### S.4.2 Yield calculation with GC-FID

Aliquots of approximately 50 μL are extracted from the reaction mixture at different times, including the beginning of the experiment, t=0 min, and are diluted using ethyl acetate before centrifugation and injection of the supernatant to the GC-FID. The corresponding yield at each stage was calculated taking into account the initial concentration of cyclohexene oxide with respect to the internal standard.

The chromatograph temperature of injection was 250ºC. The thermal programme of the oven consisted of 2 minutes at 100ºC before a 70ºC/min ramp then holding at 200ºC for a minute, followed by a second ramp of 40ºC/min until 280ºC, temperature at which it stayed for 3.5 more minutes. Using these, isolated peaks of the products were obtained at the following retention times: 4.7 min for cyclohexene oxide, 5.4 min for aniline, 6.7 min for *n*-Dodecane and 9.8 min for the product 2-(phenylamino)cyclohexan-1-ol (**3**).

**S.4.3 First order kinetic fitting for cyclohexene oxide opening with aniline using MUV-101(Fe) as catalyst**

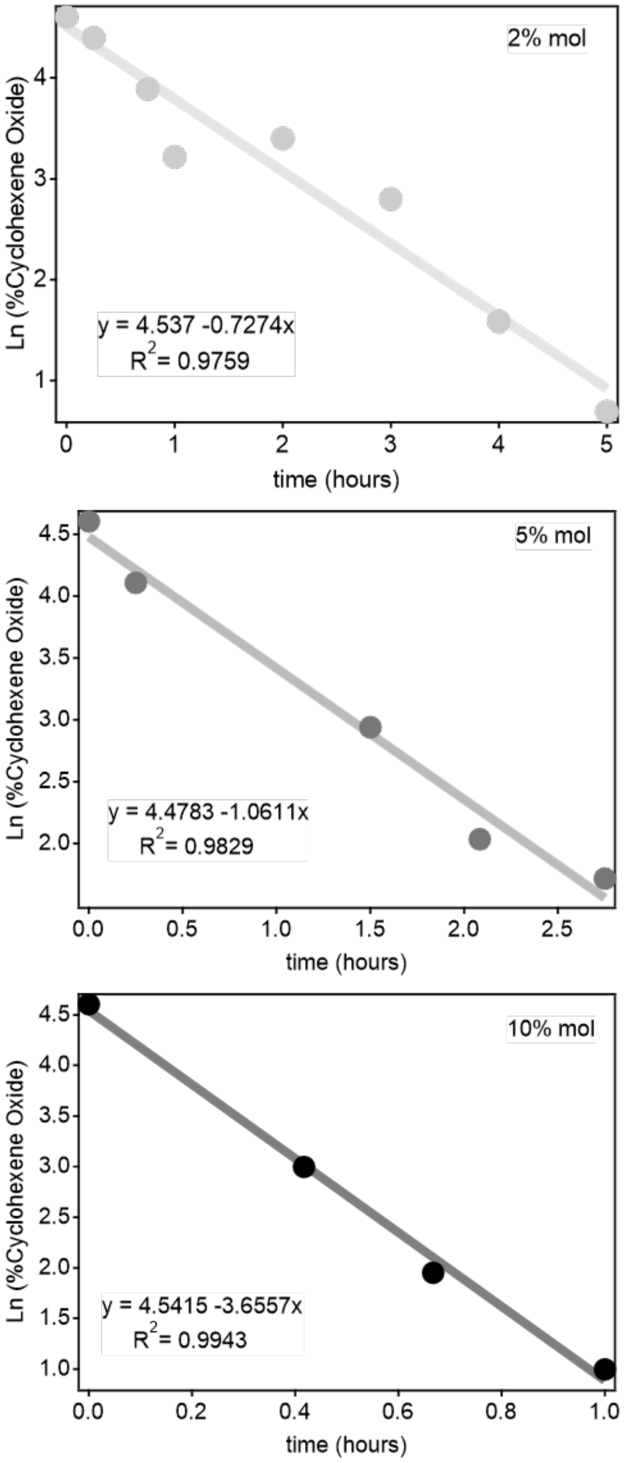


**Figure S7.** First order fitting for different catalytic loadings: 2, 5 and 10% mol MUV-101(Fe). The initial rate constant was calculated to be 0.72, 1.1 and 3.6 $h^{-1}$ for 2, 5 and 10% catalyst loading respectively.

## S.5 POISONING OF THE CATALYST

To evaluate the different contributions of each type of acidity towards the catalytic total activity, poisoning tests were carried out. To do so, two different molecules were chosen as probes for each acidic centre. In particular, following the methods described in the literature, 2,6-Lutidine[5] and pyridine[6] were chosen as molecules to specifically interact with Brønsted and Lewis acidic sites respectively thus blocking its catalytic activity.

Experimentally, 100 mg of MUV-101(Fe) were activated under vacuum at 150ºC overnight. Then, to block the Lewis acidic sites, 5 mL of pyridine were added under an Ar atmosphere. The mixture was left undisturbed for 12 hours and the supernatant was removed with a cannula. The material was activated at 110ºC and vacuum overnight. As previously reported,[6] with this activation protocol, pyridine stays coordinated only to Lewis acidic sites.

In order to evaluate the effect of the Brønsted acidic sites only, to the activated MUV-101(Fe), 5 mL of 2,6-lutidine were added. After being left still for 12 hours, the supernatant was removed and the solid was activated at 150ºC and vacuum overnight to remove any non-coordinated 2,6-lutidine.

Powder X-Ray Diffractograms and Nitrogen adsorption at 77 K were measured for both solids. The results are shown in **Figure S8** and **Figure S9,** confirming minimum impact to the structure or porosity of the solid after this treatment.

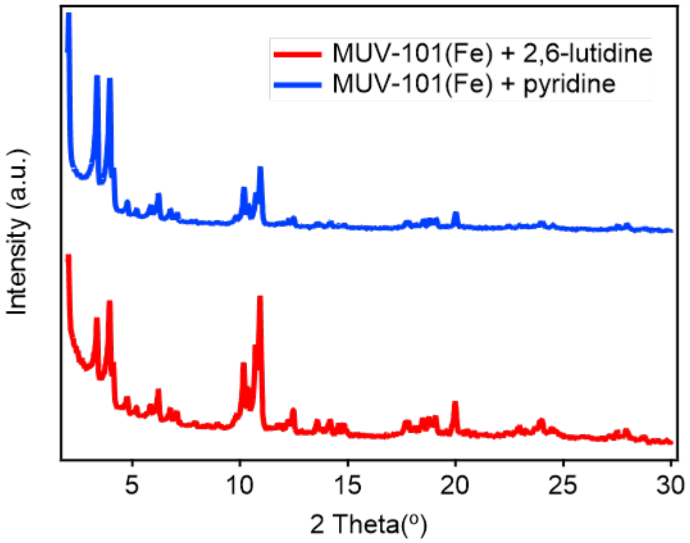


**Figure S8.** Maintenance of crystallinity of MUV-101(Fe) upon treatment with 2,4-Lutidine and pyridine.

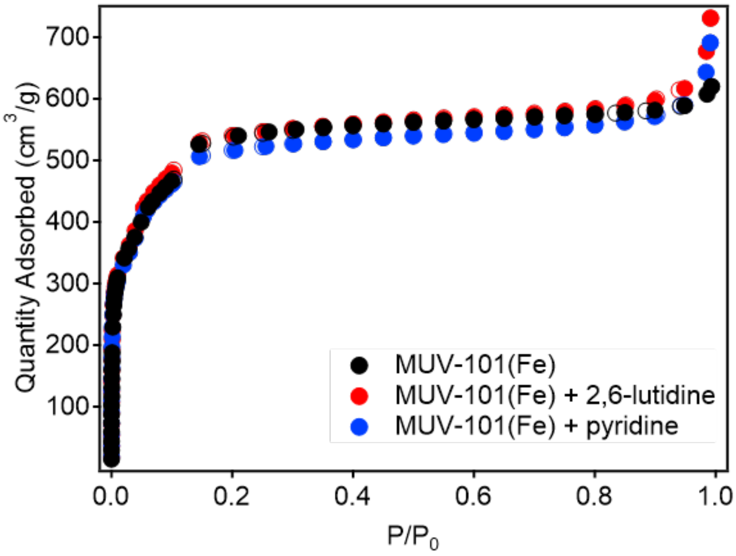


**Figure S9.** Maintenance of porosity of MUV-101(Fe) upon treatment with 2,4-Lutidine and pyridine.

## S.6 COMPARISON WITH REPRESENTATIVE MATERIALS

**Table S3.** Comparison of the catalytic activity of different MOFs reported for the Epoxide Ring-Opening reaction with amines.

| Material | Reagents | % mol | Time (h) | Yield (%) | Conditions | Ref. |
|---|---|---|---|---|---|---|
| Hf-NU-1000 | styrene oxide, $TMSN_3$ | 4 | 12 | 95 | 55 ºC, 20 eq. $TMSN_3$ | [7] |
| Zr-UiO-66-thiophene-2-carboxamido | cyclohexene oxide, aniline | 1 | 12 | 97 | neat, r.t. | [8] |
| (*R*)-CuMOF-1 | cyclohexene oxide, aniline | 10 | 48 | 86 | $CH_3Cl_3$, 50 ºC | [9] |
| UiO-68-Cr | stilbene oxide, aniline | 10 | 24 | 86 | $N_2$ atm., DCM, r.t. | [10] |
| Cu-MOF | cyclohexene oxide, aniline | 9 | 4 | 32 | excess aniline, r.t. | [11] |
| MST-1 (Zn) | propylene oxide, *N*-Methylaniline | 6 | 14 | 87 | epoxide excess, molecular sieves, 40 ºC | [12] |
| MST-2 (Co-Zn) | cyclohexene oxide, aniline | 6 | 12 | 65 | molecular sieves, 40 ºC | [12] |
| MST-1 (Co-Zn) | cyclohexene oxide, aniline | 6 | 12 | 35 | molecular sieves, 40 ºC | [12] |
| ZrOTf-BTC | cyclohexene oxide, aniline | 1 | 2 | 75 | DCM, 25 ºC | [13] |
| ZrOTf-BTC | cyclohexene oxide, aniline | 5 | 2 | 100 | DCM, 25 ºC | [13] |
| MUV-101(Fe) | cyclohexene oxide, aniline | 2 | 2 | 75 | neat, 30 ºC | *This work* |
| MUV-101(Fe) | cyclohexene oxide, aniline | 5 | 2 | 92 | neat, 30 ºC | *This work* |

## S.7 STABILITY AND RECYCLABILITY OF MUV-101(M) AND MIL-100(M) AS CATALYST

### S.7.1 PXRD of MUV-101(M) after catalysis

After 24 hours, the solid was separated through centrifugation from the reaction media, thoroughly washed with ethyl acetate and diethyl ether and let dry in air. The Powder X-ray Diffractograms of the used materials confirm the stability of them in the reaction conditions.

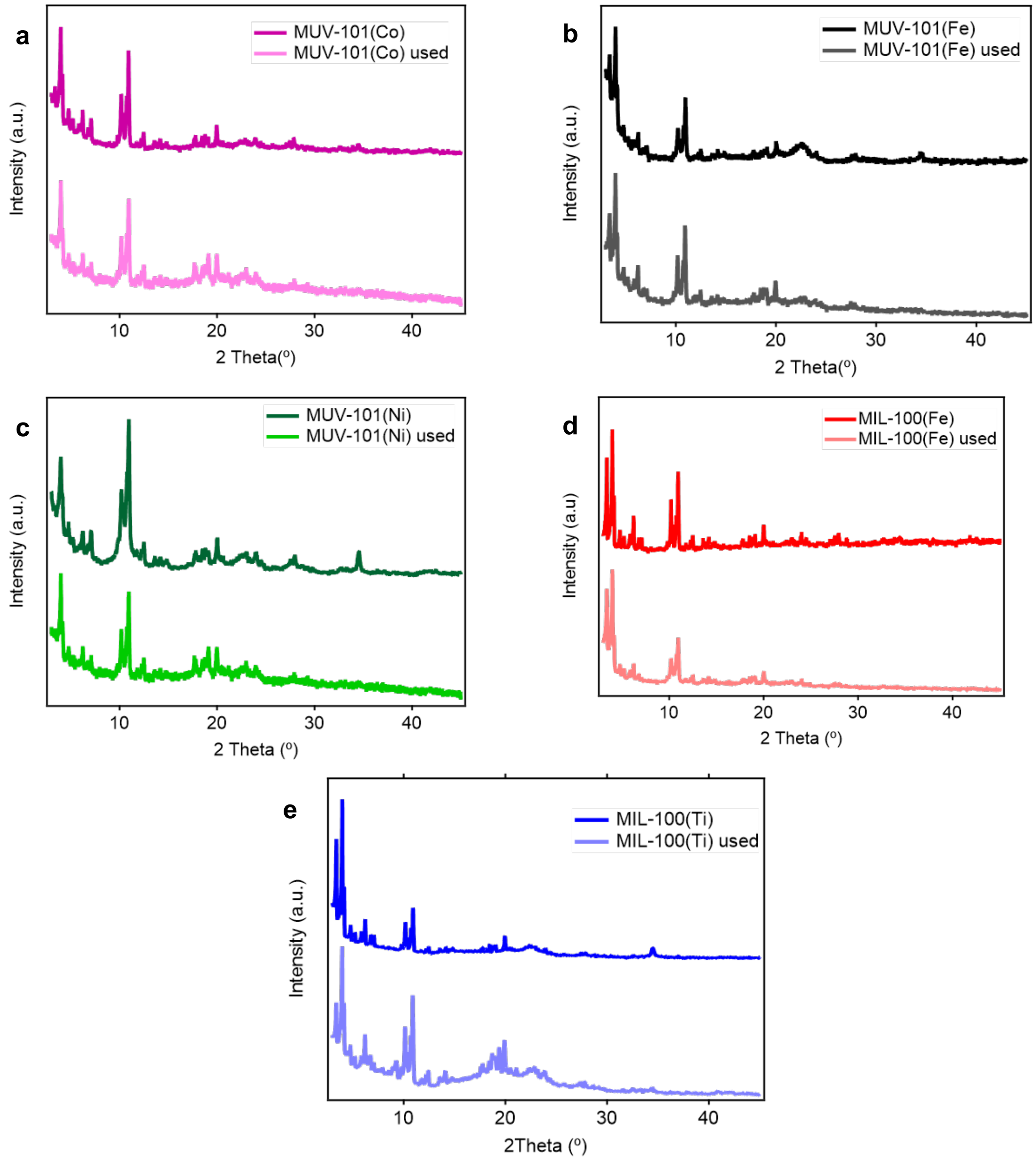


**Figure S10.** Comparison of the PXRD of the pristine and the material after catalysis for MUV-101(Co) (a), MUV-101(Fe) (b), MUV-101(Ni) (c), MIL-100(Fe) (d) and MIL-100(Ti) (e).

## S.7.2 Le Bail Refinement of used MUV-101(Fe)

To carry out the refinement, the Powder X-Ray Diffraction pattern was collected for polycrystalline samples using a 0.5 mm glass capillary mounted and aligned in a PANalytical Empyrean diffractometer (Bragg-Brentano geometry) using copper radiation (Cu $K_\alpha$ $\lambda$ = 1.5418 A) with an X'Celerator detector, operating at 40 mA and 45 kV. Profiles were collected by using a Soller Slit of 0.02° and a divergence slit of 1/4 at room temperature in the angular range 2° < 2θ < 80°with a step size of 0.013°.

Le Bail refinements were carried out with the FULLPROF software package using MIL-100(Fe) as starting parameters. The refined cell parameters obtained are the following: Fd-3m, a = b = c = 73.26245 Å, α = β = γ = 90º, $R_p$ = 2.11 %, $R_{wp}$ = 3.24 %, $R_{exp}$ = 1.89 %, Chi2 = 2.95, GoF = 1.72. The agreement between the experimental and the predicted diffractogram of used MUV-101(Fe) discards any change of its structure upon reaction.

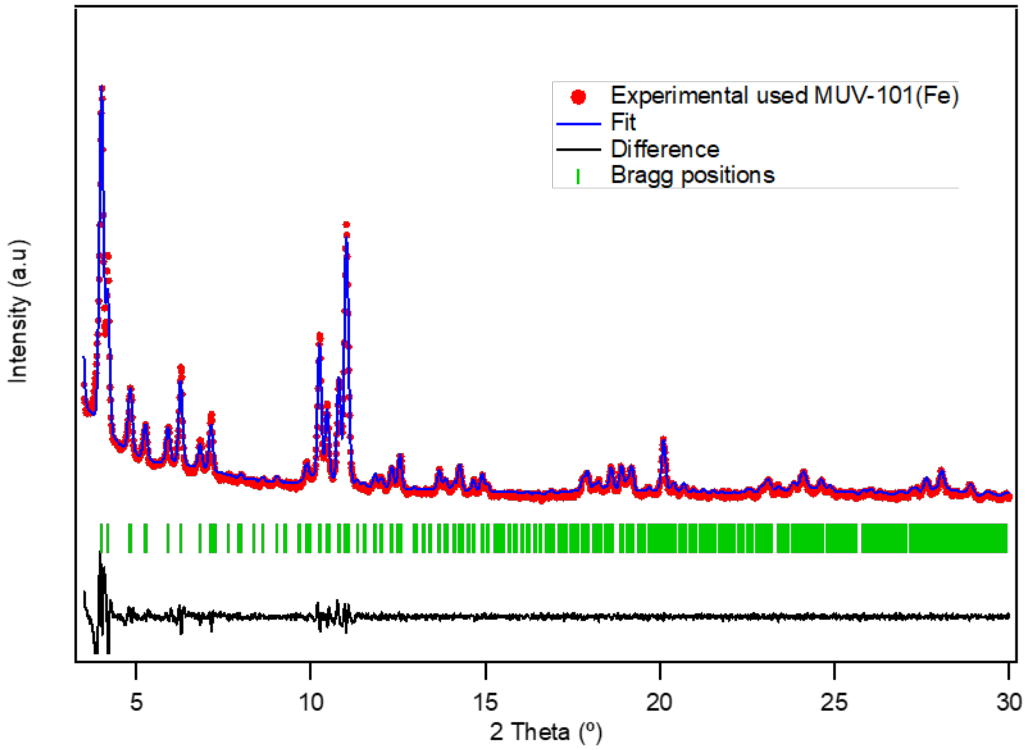


**Figure S11.** Experimental (red dots), calculated (blue line), difference plot [(Iobs−Icalc)] (black line, bottom panel) and Bragg positions (green ticks, bottom panel) for the Le Bail refinement of experimental diffraction data of MUV 101(Fe) after 4 catalytic cycles.

### S.7.3 SEM images of the used materials

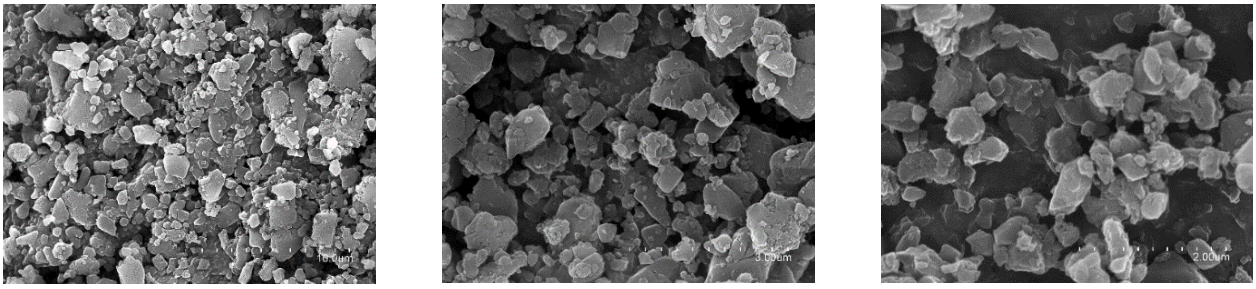


**Figure S12.** SEM images of MIL-100(Fe) after 10 hours in the reaction conditions (80 μL of cyclohexene oxide and 80 μL of aniline).

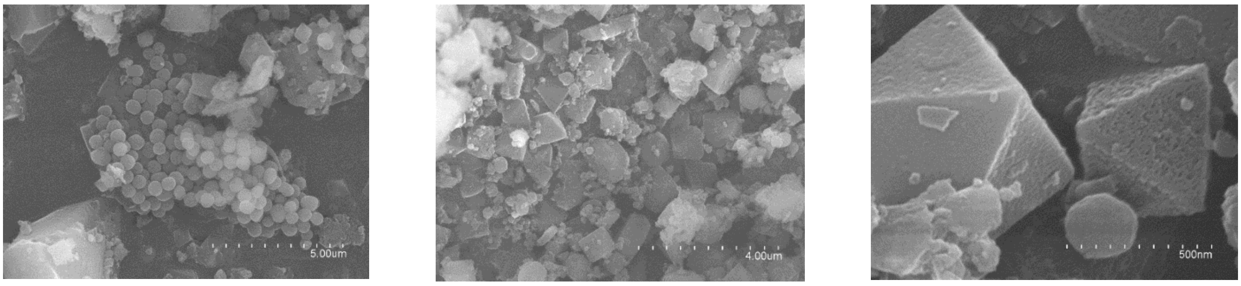


**Figure S13.** SEM images of MIL-100(Ti) after 10 hours in the reaction conditions (80 μL of cyclohexene oxide and 80 μL of aniline).

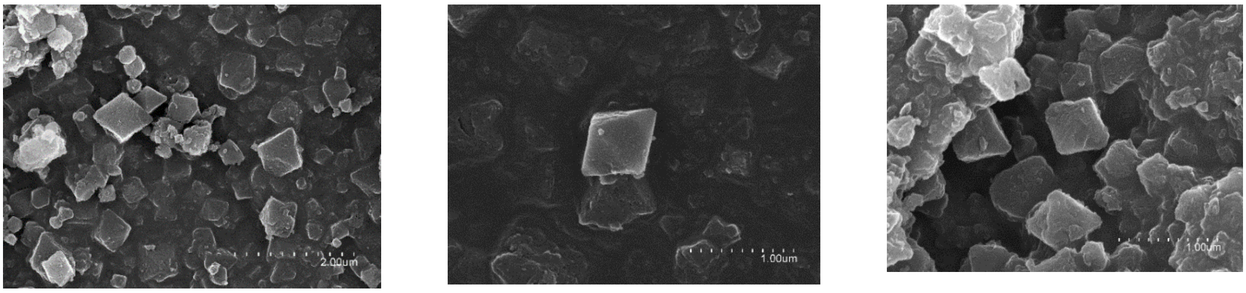


**Figure S14.** SEM images of MUV-101(Fe) after 10 hours in the reaction conditions (80 μL of cyclohexene oxide and 80 μL of aniline).

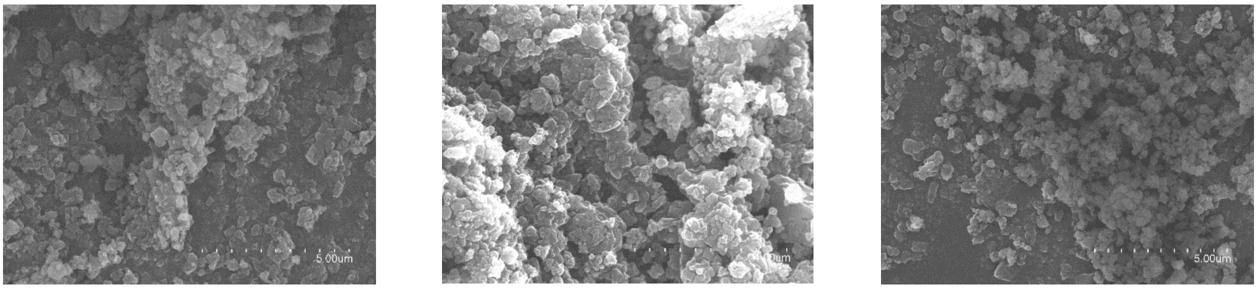


**Figure S15.** SEM images of MUV-101(Ni) after 10 hours in the reaction conditions (80 μL of cyclohexene oxide and 80 μL of aniline).

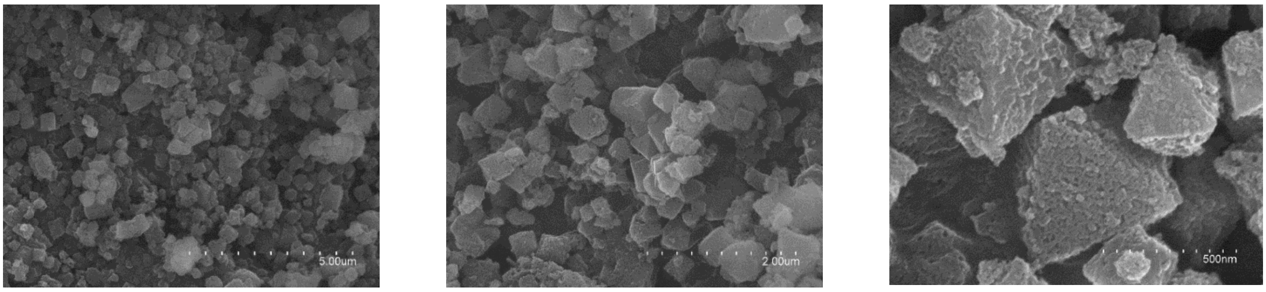


**Figure S16.** SEM images of MUV-101(Co) after 10 hours in the reaction conditions (80 μL of cyclohexene oxide and 80 μL of aniline)

### S.7.4 $N_2$ Adsorption of MUV-101(Fe) after catalysis

Gas adsorption measurements were recorded on a Micromeritics 3Flex apparatus. Prior to analysis, samples were degassed overnight at 150 ºC and $10^{-6}$ Torr. The nitrogen adsorption of the material and the value of BET surface area, were maintained through several cycles, as derives from **Figure S17** and **Table S4**, respectively.

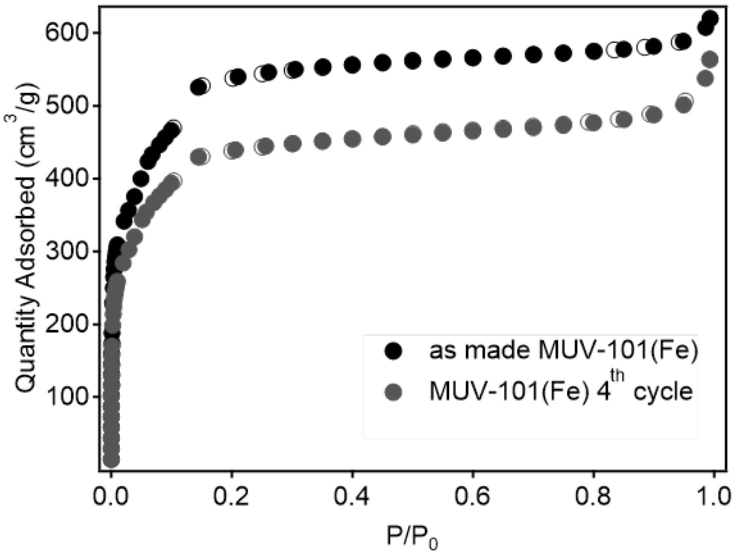


**Figure S17.** $N_2$ adsorption isotherm for the as made MUV-101(Fe) and the MUV-101(Fe) after 4 subsequent cycles of catalysis. The small decrease of $N_2$ uptake at high relative pressures is possibly due to the occlusion of reaction products during the catalytic cycles.

**Table S4.** Values of BET surface area obtained for the as made MUV-101(Fe) and after the 4$^{th}$ catalytic cycle.

| BET surface area as made ($m^2/g$) | BET surface area after 4$^{th}$ cycle ($m^2/g$) |
|---|---|
| 1972.7± 20.0 | 1570.4 ± 5.4 |

### S.7.5 Recyclability tests for MUV-101(Fe)

After each catalytic cycle, the material was washed twice with ethyl acetate and then with diethyl ether to remove any organic substance left in the pore. This washed material was recovered and activated using a similar methodology than that of the fresh one. The evaluation of the recyclability was carried out by comparing the yield obtained in the same reaction conditions after (2% mol MOF and 0.8 mmol reaction scale, neat for 10 hours). As demonstrated in **Figure S18**, no significant change was found in its catalytic activity, in agreement with the maintenance of the MOF structure previously observed.

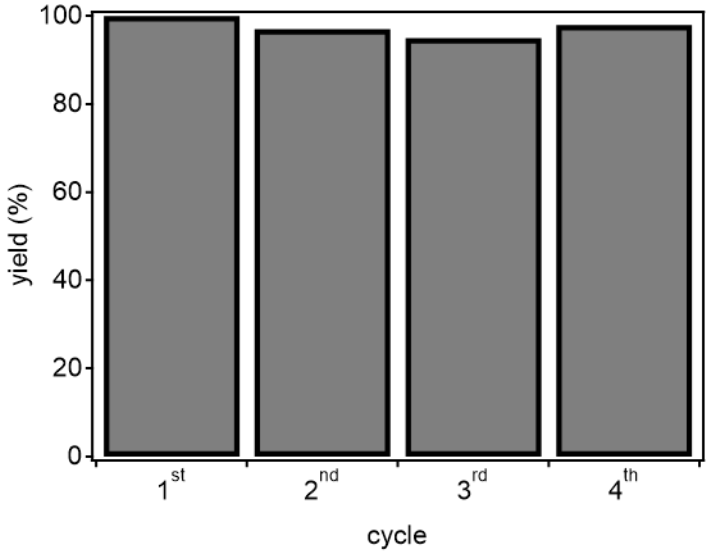


**Figure S18.** Yield obtained in the 4 subsequent catalytic cycles of MUV-101(Fe) at 2% mol MOF, 0.8 mmol reaction scale and 30º C after 10 hours.

### S.7.6 ICP and Hot-Filtration Test

**ICP**

ICP-MS was performed to the supernatant of the reaction after 24 hours. Following the general procedure for the catalysis, 80 µL of cyclohexene oxide (0.80 mmol), 80 µL of aniline (0.85 mmol) and 80 µL of dodecane (0.35 mmol) were added to a vial containing 10 mg of previously activated MUV-101(Fe). The reaction was left stirring at 30ºC under neat conditions for 24 hours. After this time, in order to study the catalyst stability in the reaction media, the supernatant was separated through centrifugation at 8000 rpm during 10 min. The concentration in mg/L of the different metals found using this technique are presented in **Table S4**.

**Table S5.** Concentration (mg/L) of the different metals in solution determined by ICP-MS of MUV-101(M) and MIL-100(M) in the reaction conditions after 24 hours. Regarding MUV-101(Fe), this value would account for a negligible framework decomposition of near 0.27%.

| MIL-100(Ti) | MUV-101(Co) | | MUV-101(Ni) | | MUV-101(Fe) | | MIL-100(Fe) |
|---|---|---|---|---|---|---|---|
| [Ti] | [Ti] | [Co] | [Ti] | [Ni] | [Ti] | [Fe] | [Fe] |
| 48.2 ± 0.8 | 93 ± 3 | 196 ± 3 | 2.22 ± 0.002 | 8.65 ± 0.09 | 10.957± 0.13 | 16.84 ± 0.14 | 2.45 ± 0.03 |

### Hot-Filtration Test

A Hot-Filtration Test (HFT) was carried out to confirm the heterogeneous nature of the reaction with MUV-101(Fe), ruling out the possibility of leached metal ions acting as catalytic centres. In order to conduct the experiment, the reaction was carried out following the general procedure for 2% MOF loading (80 µL of aniline, cyclohexene oxide and *n*-dodecane at 30ºC). The mixture was stirring for 45 min. After that time, the supernatant of the reaction was separated through centrifugation at 8000 rpm during 10 min. Then, the solution was filtered and placed in a new vial and its evolution followed through GC-FID at different times. As can be seen from **Figure S19**, the yield of the desired product (**3**) did not evolve over time in the absence of catalyst.

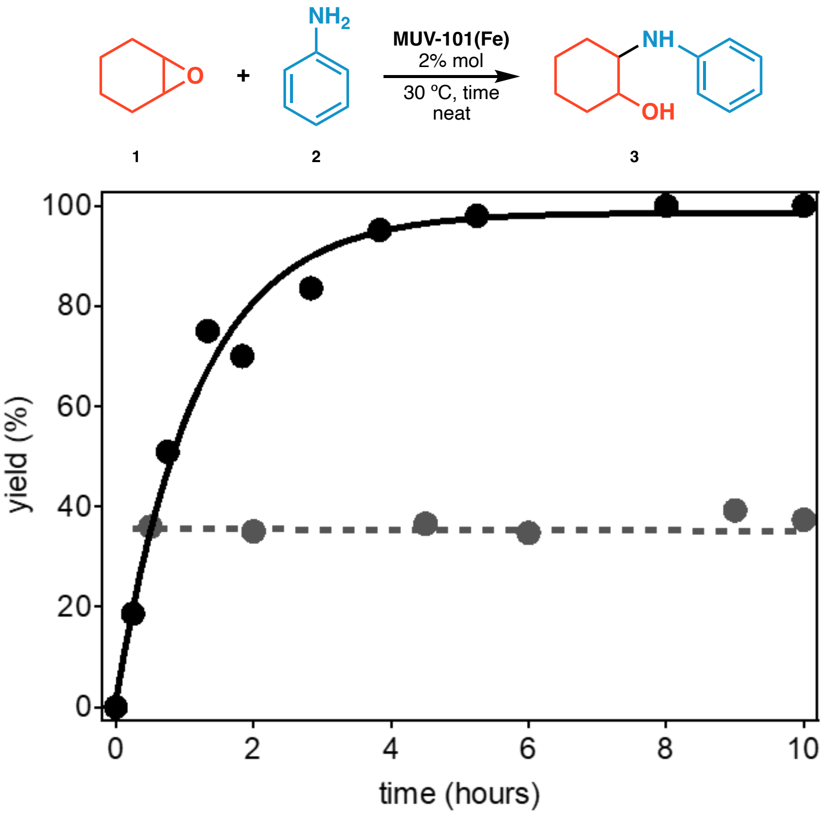


**Figure S19.** Hot-Filtration Test of the reaction of cyclohexene oxide with aniline using MUV-101(Fe) as a catalyst. Dotted-grey line represents the supernatant of the reaction after separation of the solid catalyst.

## S.8. SCOPE OF THE REACTION

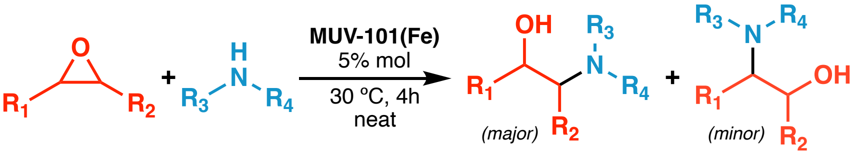


Following the general procedure for the 5% MOF loading (*see section S.4.1*), 0.34 mmol, (1 equiv.) of the corresponding amine, 0.32 mmol, (1 equiv.) of the corresponding oxirane and 80 µL of *n*-Dodecane as internal standard were added to 2 mL vial containing 10 mg of activated MUV-101(Fe) (5% mol MOF). The mixture was stirring during 4h at 30ºC. Subsequently, the crude was dissolved in a minimal amount of DCM and purified by flash column chromatography using hexane and ethyl acetate as eluent.

For GC-FID yields, after 2/4 hours, an aliquot was extracted from the reaction media, diluted with ethyl acetate and centrifuged before being injected in the Gas Chromatograph in order to obtain the yield.

**Table S6.** Different epoxides and amines used to explore the scope of the reaction.

| Epoxides | Amines |
|---|---|
| 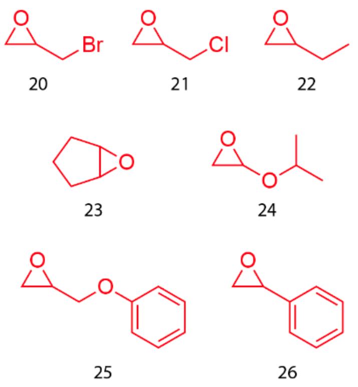 | 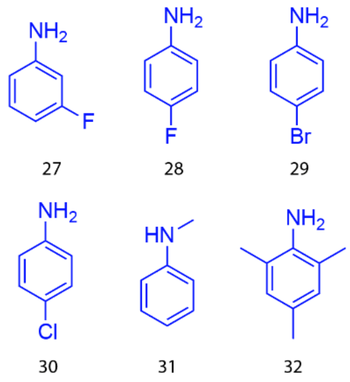 |

For the synthesis of the natural product, propranolol (**19**), also named 1-(isopropylamino)-3-(naphthalen-1-yloxy)propan-2-ol, the reaction conditions were a 0.36 mmol scale, with 10 mg of activated MUV-101(Fe) (5% catalyst loading) and stirring at 50ºC for 24 hours.

The synthesis of propranolol was also scaled up to a 3.6 mmol scale, maintaining the catalyst loading of activated MUV-101(Fe) fixed at 5% (100 mg of MOF). The reaction was left stirring at 50ºC for 24 hours and the net conversion reached near 90%.

**2-(phenylamino)cyclohexan-1-ol (3)** was obtained using 0.8 mmol of cyclohexene oxide (**1**) and aniline (**2**), 10 mg of activated MUV-101(Fe), stirring at 30ºC for 5 hours. This product was isolated by flash column chromatography (hexane: ethyl acetate; 7:3) as a yellow solid; Yield: 96%. NMR data of the compound **3** match with the previously reported in the literature.[13] $^1$H NMR (300 MHz, $CDCl_3$) δ: 7.24 – 7.13 (m, 2H), 6.81 – 6.69 (m, 3H), 3.37 (td, *J* = 9.8, 4.4 Hz, 1H), 3.15 (ddd, *J* = 11.1, 9.3, 4.0 Hz, 1H), 2.19 – 2.05 (m, 2H), 1.84 – 1.66 (m, 2H), 1.49 – 1.23 (m, 3H), 1.16 – 0.98 (m, 1H).

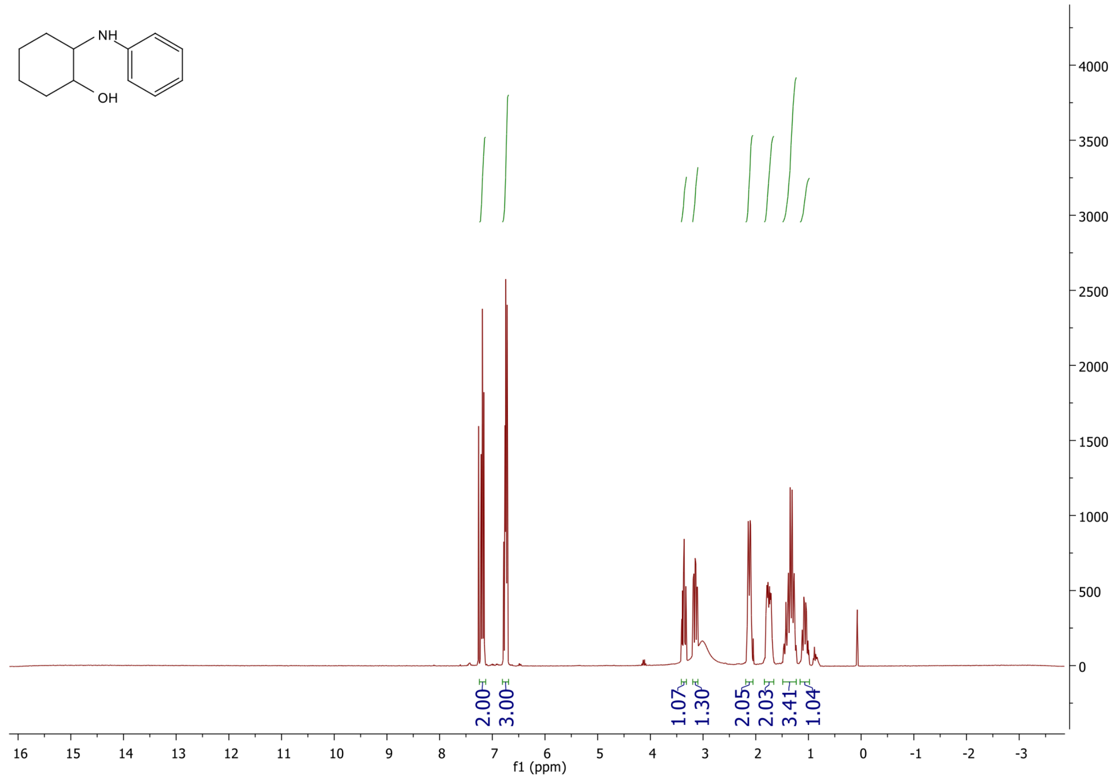

**1-bromo-3-(phenylamino)propan-2-ol (4):** Following the general procedure described above, reaction of the epoxide **20** with amine **2** afforded the title compound **4** as a dark oil. Purification by flash column chromatography (hexane: ethyl acetate; 9:1). Yield: 87%. NMR data of the compound **4** match with the previously reported in the literature.[14] $^1$H NMR (300 MHz, $CDCl_3$) δ: 7.25 – 7.17 (m, 2H), 6.83 – 6.67 (m, 3H), 4.13 – 4.03 (m, 1H), 3.61 – 3.46 (m, 2H), 3.41 (dd, *J* = 13.3, 4.4 Hz, 1H), 3.25 (dd, *J* = 13.3, 7.3 Hz, 1H).

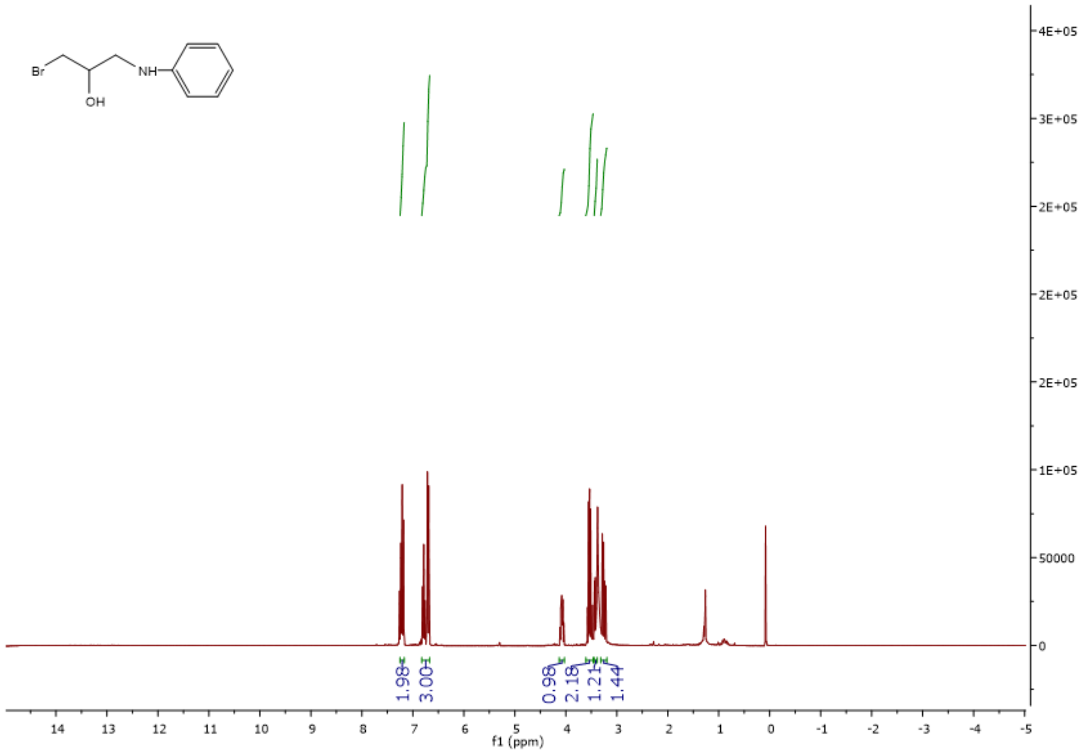

**1-chloro-3-(phenylamino)propan-2-ol (5):** Following the general procedure described above, reaction of the epoxide **21** with amine **2** afforded the title compound **5** as a yellow solid. Purification by flash column chromatography (hexane: ethyl acetate; 7:3). Yield: 99%. NMR data of **5** match with the previously reported in the literature.[15] $^1$H NMR (300 MHz, $CDCl_3$) δ: 7.17 – 7.08 (m, 2H), 6.73 – 6.66 (m, 1H), 6.64 – 6.57 (m, 2H), 4.02 (ddd, *J* = 11.1, 6.2, 4.6 Hz, 1H), 3.68 – 3.51 (m, 2H), 3.33 (dd, *J* = 13.3, 4.5 Hz, 1H), 3.18 (dd, *J* = 13.3, 7.1 Hz, 1H).

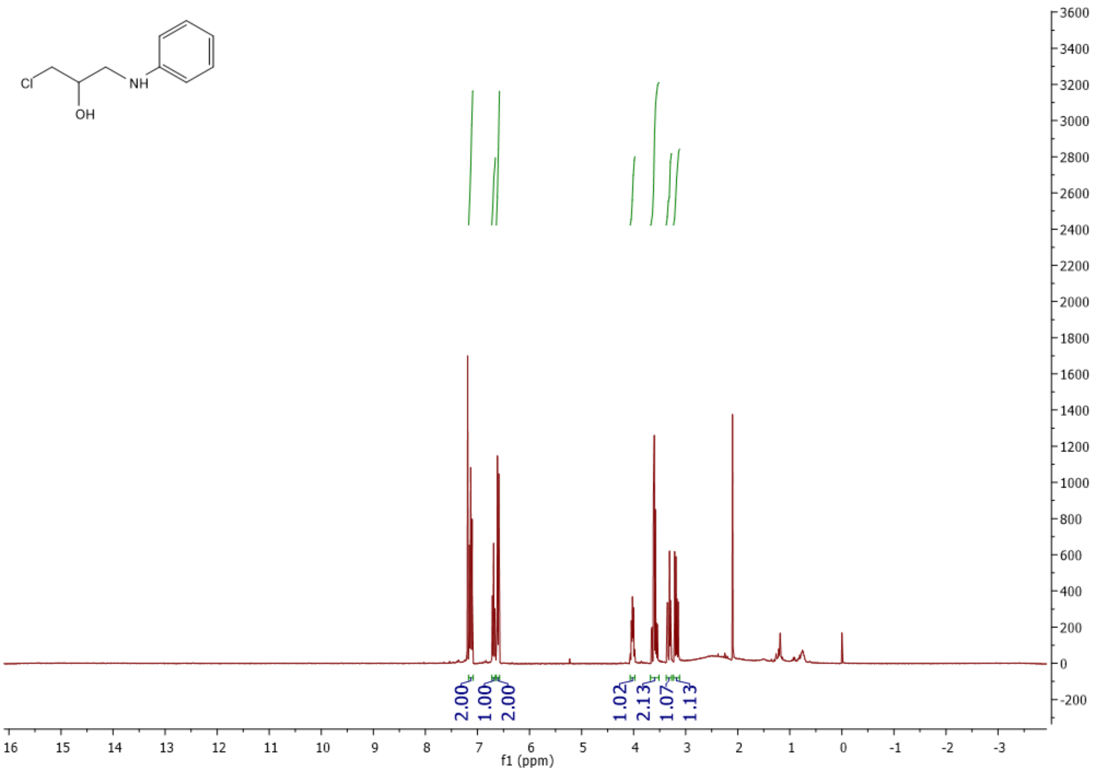

**1-(phenylamino)butan-2-ol (6):** Following the general procedure described above, reaction of the epoxide **22** with amine **2** afforded the title compound **6** as a yellow oil. Purification by flash column chromatography (hexane: ethyl acetate; 8:2).; Yield: 99%. NMR data of **6** match with the previously reported in the literature. [16] $^1$H NMR (300 MHz, $CDCl_3$) δ: 7.23 – 7.15 (m, 2H), 6.74 (t, *J* = 7.3 Hz, 1H), 6.67 (d, *J* = 8.0 Hz, 2H), 3.85 – 3.71 (m, 1H), 3.28 (dd, *J* = 12.8, 2.9 Hz, 1H), 3.02 (dd, *J* = 12.8, 8.6 Hz, 1H), 1.67 – 1.48 (m, 2H), 1.02 (t, *J* = 7.4 Hz, 3H).

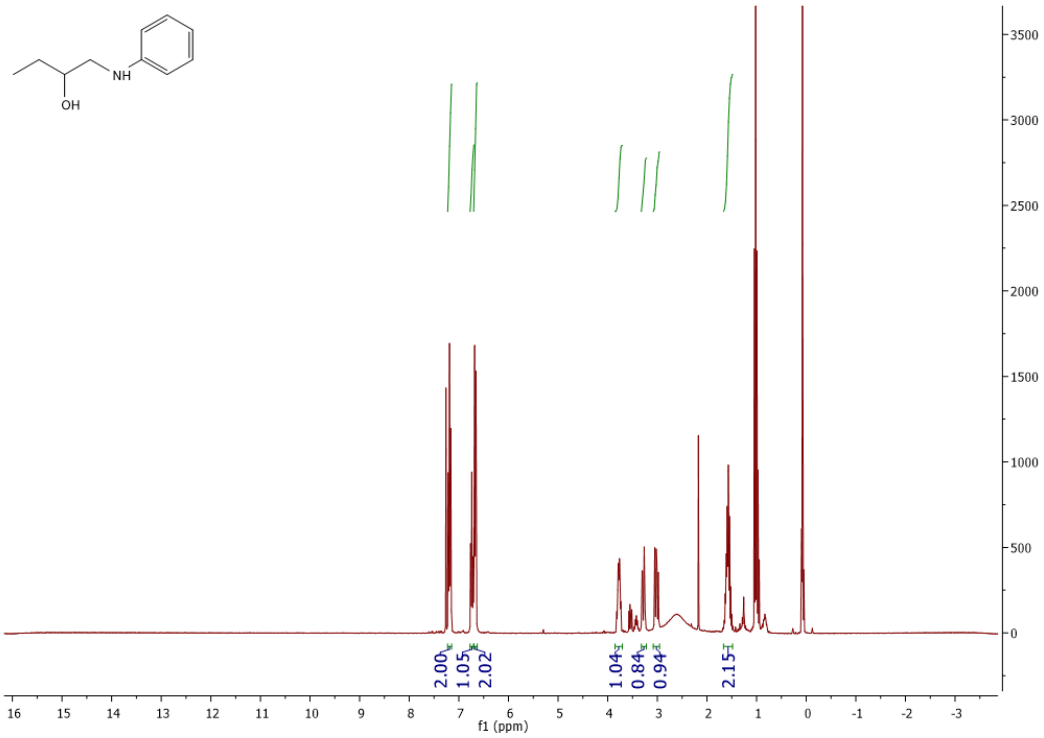

**2-(phenylamino)cyclopentan-1-ol (7):** Following the general procedure described above, reaction of the epoxide **23** with amine **2** afforded the title compound **7** as a brown solid. Purification by flash column chromatography (hexane: ethyl acetate; 7:3). Yield: 94%. NMR data of **7** match with the previously reported in the literature.[13] $^1$H NMR (300 MHz, $CDCl_3$) δ: 7.24 – 7.13 (m, 2H), 6.78 – 6.62 (m, 3H), 4.06 (dt, $J$ = 6.0, 4.5 Hz, 1H), 3.66 – 3.57 (m, 1H), 2.36 – 2.19 (m, 1H), 2.09 – 1.92 (m, 1H), 1.92 – 1.70 (m, 2H), 1.72 – 1.56 (m, 1H), 1.49 – 1.33 (m, 1H).

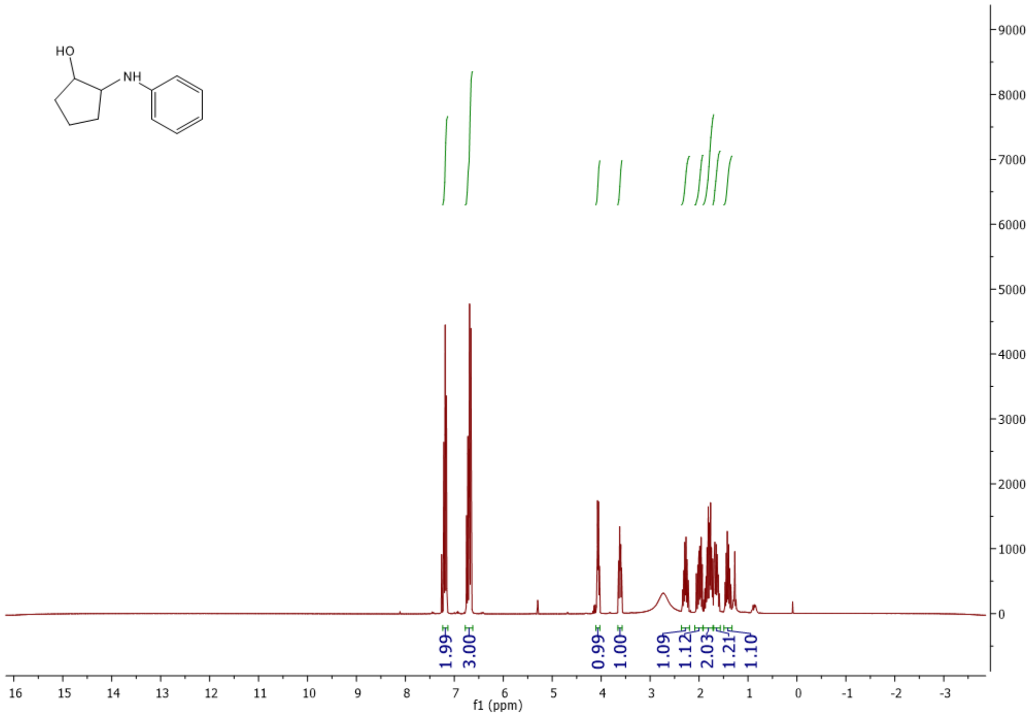

**1-isopropoxy-3-(phenylamino)propan-2-ol (8):** Following the general procedure described above, reaction of the epoxide **24** with amine **2** afforded the title compound **8** as a brown solid. Purification by flash column chromatography (hexane: ethyl acetate; 9:1). Yield: 91%. NMR data of **8** match with the previously reported in the literature.[17] $^1$H NMR (300 MHz, $CDCl_3$) δ: 7.18 (t, *J* = 7.9 Hz, 2H), 6.79 – 6.58 (m, 3H), 4.05 – 3.94 (m, 1H), 3.73 – 3.50 (m, 2H), 3.46 (dd, *J* = 9.4, 6.4 Hz, 1H), 3.30 (dd, *J* = 12.7, 4.3 Hz, 1H), 3.22 – 3.09 (m, 1H), 1.19 (d, *J* = 6.1 Hz, 6H).

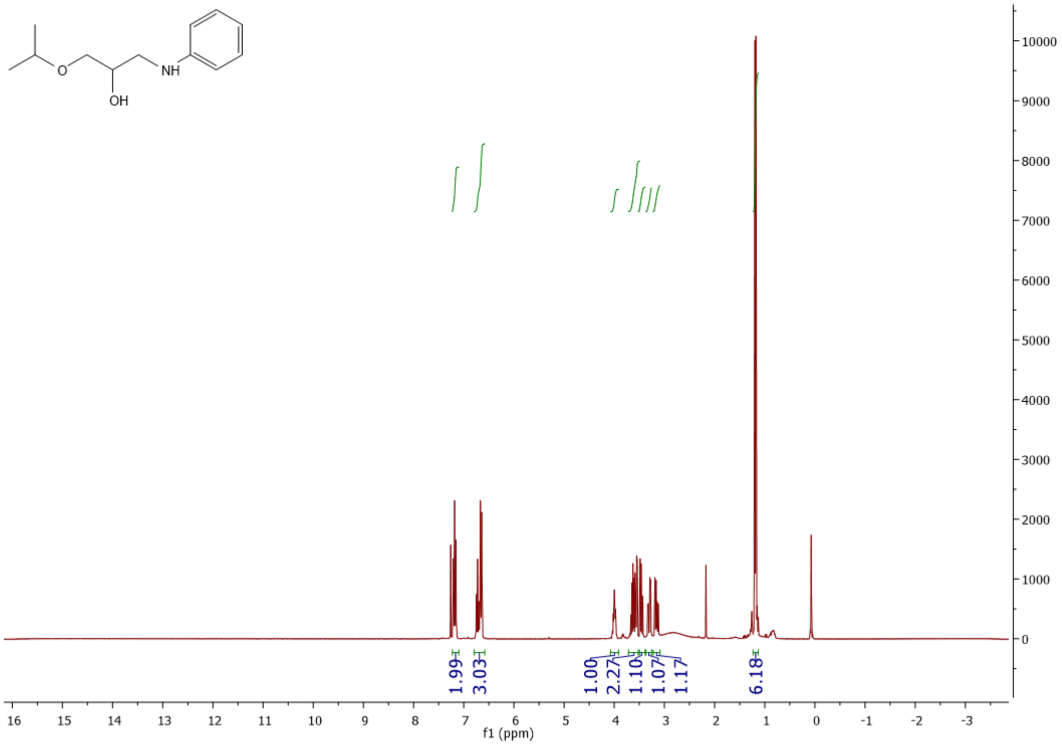

**1-phenyl-2-(phenylamino)ethan-1-ol (9):** Following the general procedure described above, reaction of the epoxide **26** with amine **2** afforded the title compound **9** as a brown solid. Purification by flash column chromatography (hexane: ethyl acetate; 7:3). Yield: 94%. NMR data of **9** match with the previously reported in the literature. [13] $^{1}$H NMR (300 MHz, $CDCl_3$) δ: 7.44 – 7.28 (m, 4H), 7.30 – 7.22 (m, 1H), 7.15 – 7.06 (m, *J* = 7.9 Hz, 2H), 6.68 (t, *J* = 7.3 Hz, 1H), 6.57 (d, *J* = 7.9 Hz, 2H), 4.50 (dd, *J* = 6.9, 4.2 Hz, 1H), 3.94 (dd, *J* = 11.1, 4.2 Hz, 1H), 3.76 (dd, *J* = 11.1, 7.0 Hz, 1H).

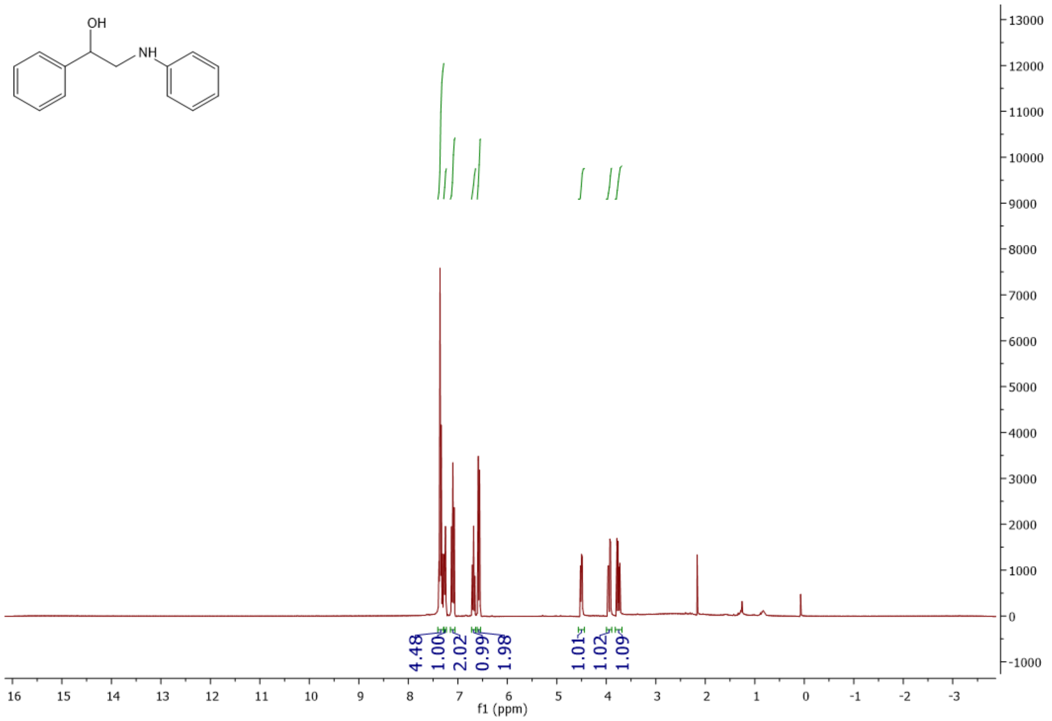

**1-phenoxy-3-(phenylamino)propan-2-ol (10):** Following the general procedure described above, reaction of the epoxide **25** with amine **2** afforded the title compound **10** as a dark solid. Purification by flash column chromatography (hexane: ethyl acetate; 9:1). Yield: 80%. NMR data of **10** match with the previously reported in the literature. [15] $^1$H NMR (300 MHz, $CDCl_3$) δ: 7.36 – 7.27 (m, 2H), 7.25 – 7.16 (m, 2H), 7.04 – 6.91 (m, 3H), 6.81 – 6.66 (m, 3H), 4.33 – 4.21 (m, 1H), 4.13 – 4.01 (m, 2H), 3.45 (dd, $J$ = 13.0, 4.4 Hz, 1H), 3.31 (dd, $J$ = 13.0, 7.1 Hz, 1H).

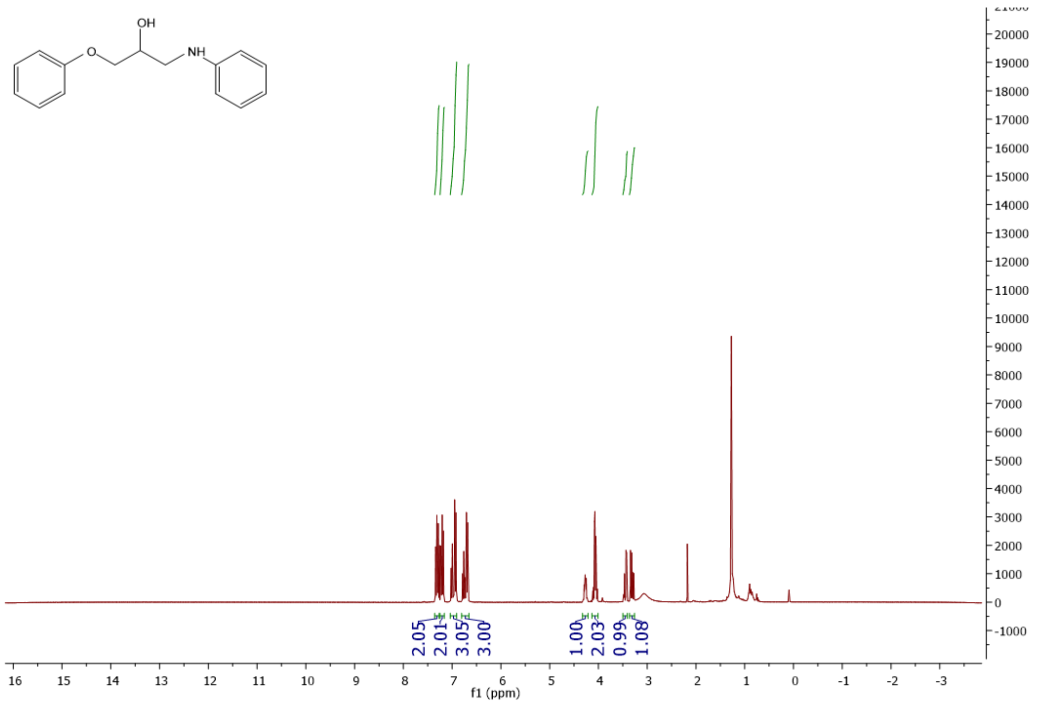

**2-((3-fluorophenyl)amino)cyclohexan-1-ol (11)**: Following the general procedure described above, reaction of the epoxide **1** with amine **27** afforded the title compound **11** as a dark oil. Purification by flash column chromatography (hexane: ethyl acetate; 7:3). Yield: 87%; $^{1}$H NMR (300 MHz, $CDCl_3$) δ: 7.14 – 7.04 (m, 1H), 6.50 – 6.37 (m, 3H), 3.36 (td, *J* = 9.8, 4.3 Hz, 1H), 3.10 (ddd, *J* = 11.1, 9.3, 4.0 Hz, 1H), 2.11 (dd, *J* = 11.0, 4.2 Hz, 2H), 1.84 – 1.64 (m, 2H), 1.49 – 1.22 (m, 3H), 1.17 – 0.96 (m, 1H). $^{13}$C NMR (75 MHz, $CDCl_3$) δ: 164.1(C) (d, *J* = 243.2 Hz), 149.6 (C) (d, *J* = 10.5 Hz), 130.4 (CH) (d, *J* = 10.2 Hz), 110.1 (CH) (d, *J* = 2.3 Hz), 104.7 (CH) (d, *J* = 21.5 Hz), 100.9 (CH), (d, *J* = 25.3 Hz), 74.43 (CH) (s), 60.1 (C), 33.3 ($CH_2$), 31.5 ($CH_2$), 24.9 ($CH_2$) (s), 24.2 $CH_2$). $^{19}$F NMR (282 MHz, $CDCl_3$) δ: -113.03 (s). Calculated for $C_{12}H_{17}FNO$ [M+1] = 210.1294; found: 210.1291.

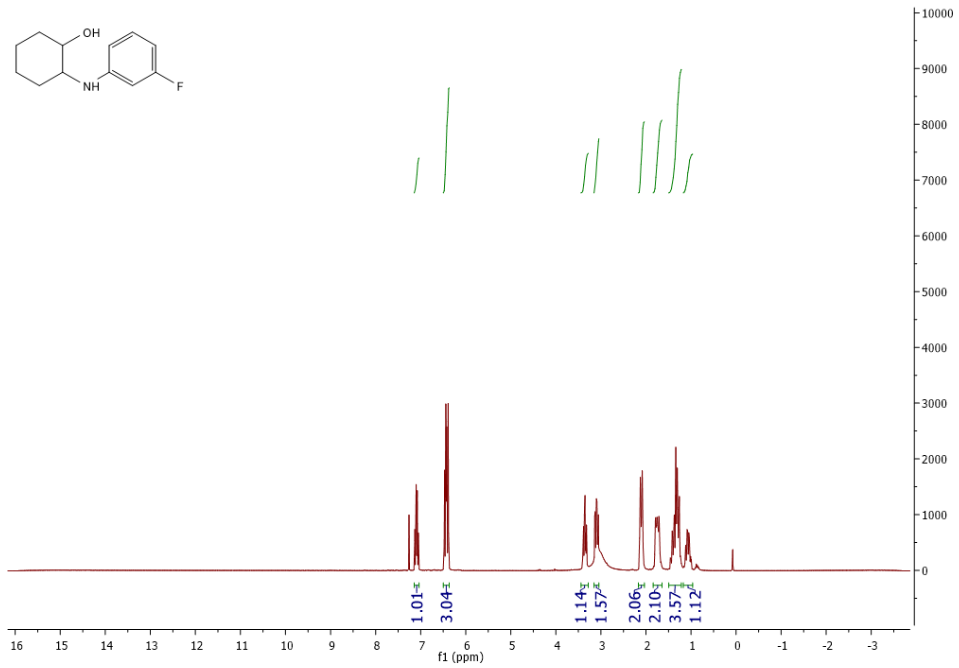


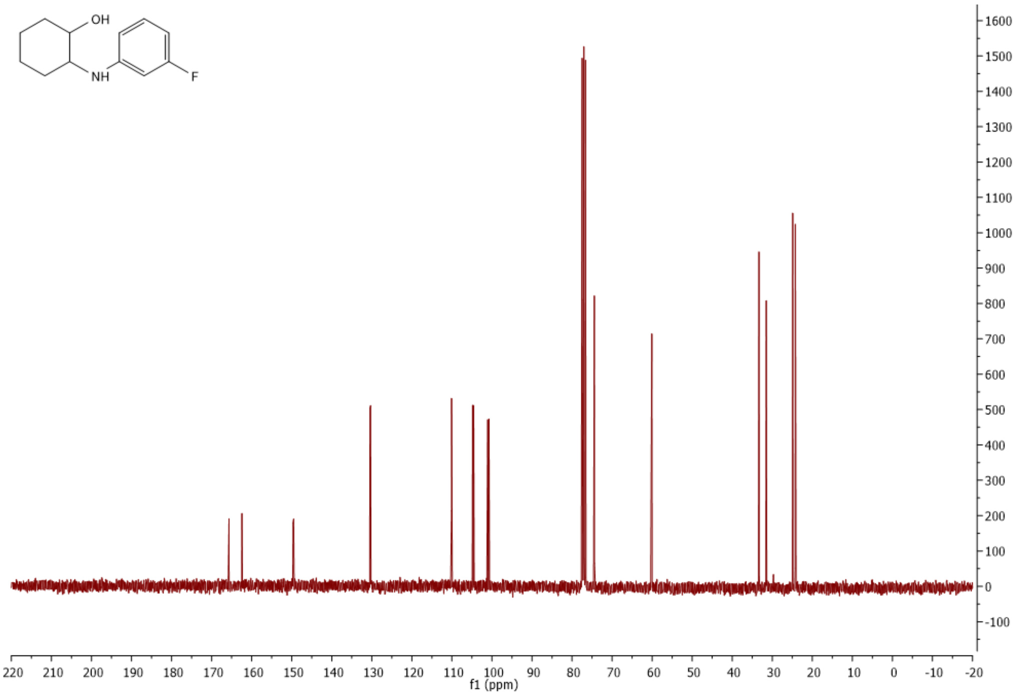

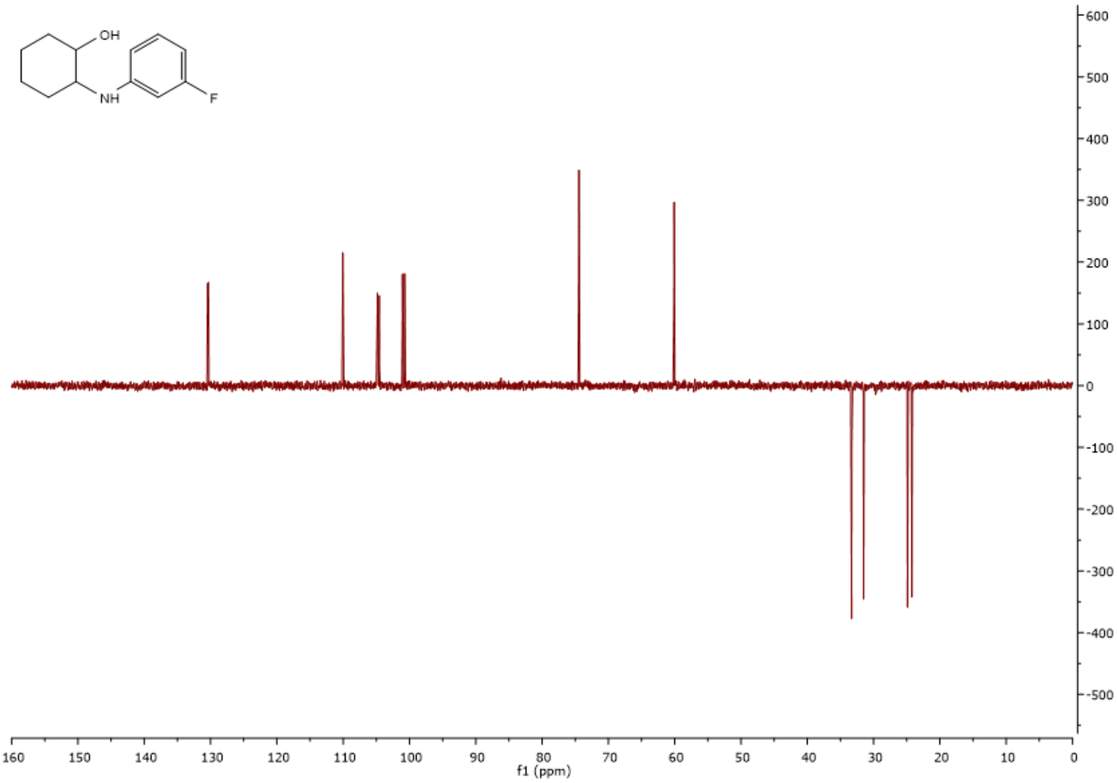

OH
NH
F
f1 (ppm)


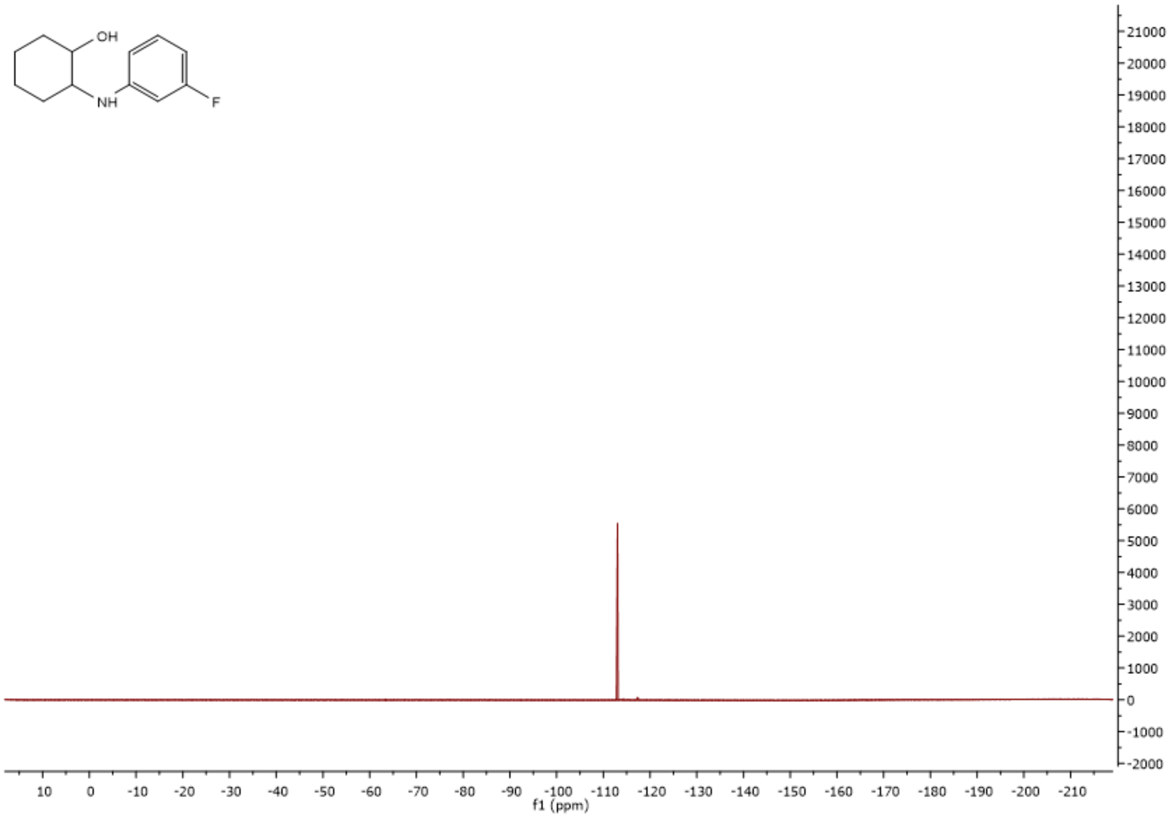

OH
NH
F
f1 (ppm)

**2-((4-fluorophenyl)amino)cyclohexan-1-ol (12):** Following the general procedure described above, reaction of the epoxide **1** with amine **28** afforded the title compound **12** as a brown solid. Purification by flash column chromatography (hexane: ethyl acetate; 7:3). Yield: 90%. NMR data of **12** match with the previously reported in the literature.[18] $^{1}H$ NMR (300 MHz, $CDCl_3$) δ: 6.88 – 6.76 (m, 2H), 6.65 – 6.53 (m, 2H), 3.28 (td, *J* = 10.2, 4.4 Hz, 1H), 2.96 (ddd, *J* = 11.2, 9.2, 4.0 Hz, 1H), 2.08 – 1.93 (m, 2H), 1.79 – 1.55 (m, 2H), 1.44 – 0.82 (m, 4H).

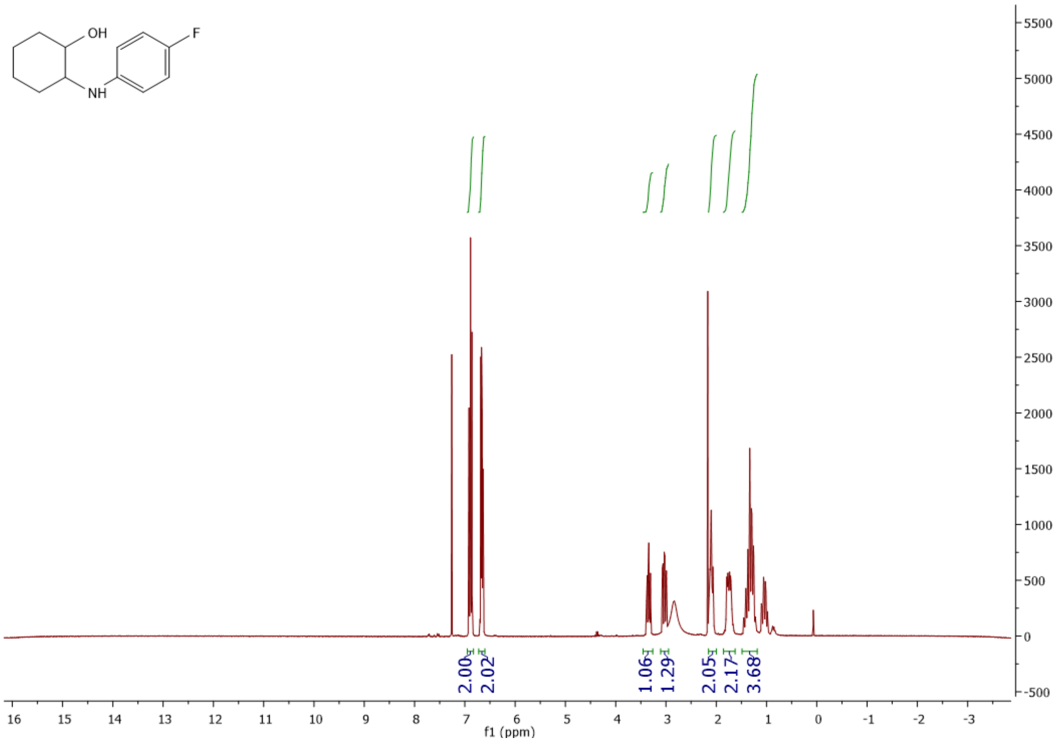

**2-((4-bromophenyl)amino)cyclohexan-1-ol (13):** Following the general procedure described above, reaction of the epoxide **1** with amine **29** afforded the title compound **13** as a brown solid. Purification by flash column chromatography (hexane: ethyl acetate; 7:3). Yield: 80%. NMR data of **13** match with the previously reported in the literature. [19] $^1$H NMR (300 MHz, $CDCl_3$) δ: 7.29 – 7.25 (m, 2H), 6.65 – 6.58 (m, 2H), 3.38 (td, *J* = 9.9, 4.4 Hz, 1H), 3.10 (ddd, *J* = 11.1, 9.2, 4.0 Hz, 1H), 2.18 – 2.04 (m, 2H), 1.84 – 1.69 (m, 2H), 1.48 – 1.22 (m, 3H), 1.16 – 1.00 (m, 1H).

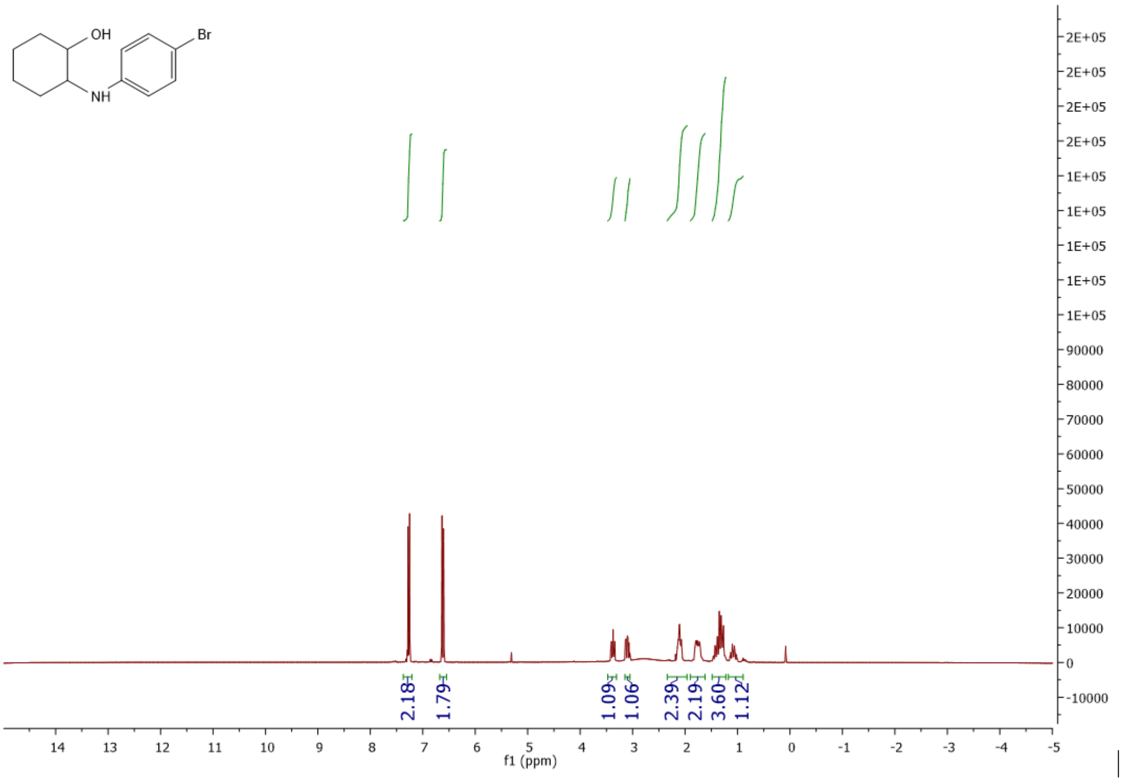

**2-((4-chlorophenyl)amino)cyclohexan-1-ol (14)**: Following the general procedure described above, reaction of the epoxide **1** with amine **30** afforded the title compound **14** as a brown solid. Purification by flash column chromatography (hexane: ethyl acetate; 7:3). Yield: 90%. NMR data of the **14** match with the previously reported in the literature.[20] $^1$H NMR (300 MHz, $CDCl_3$) δ: 7.12 (d, *J* = 8.8 Hz, 2H), 6.64 (d, *J* = 8.8 Hz, 2H), 3.44 – 3.30 (m, 1H), 3.15 – 3.03 (m, 1H), 2.15 – 2.05 (m, 2H), 1.84 – 1.65 (m, 2H), 1.43 – 1.23 (m, 3H), 1.14 – 0.97 (m, 1H).

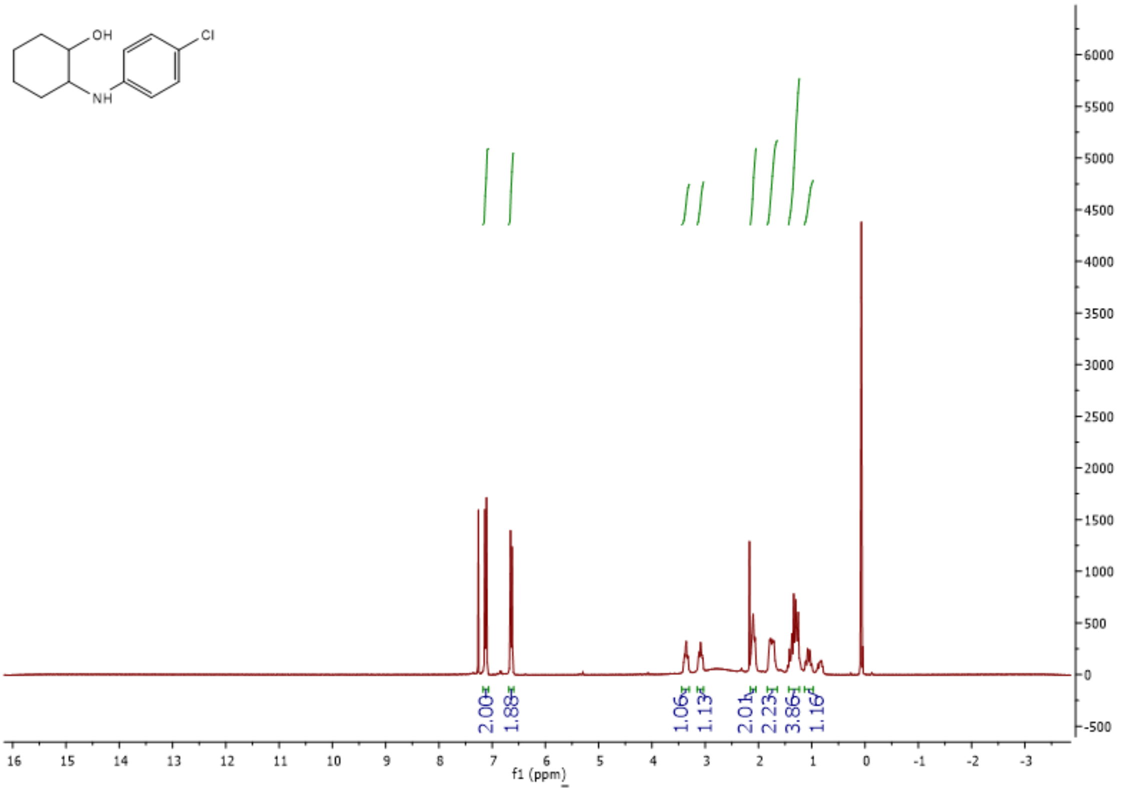

**2-(methyl(phenyl)amino)cyclohexan-1-ol (15):** Following the general procedure described above, reaction of the epoxide **1** with amine **31** afforded the title compound **15** as a yellow oil. Purification by flash column chromatography (hexane: ethyl acetate; 8:2). Yield: 75%. NMR data of **15** match with the previously reported in the literature. [13] $^1$H NMR (300 MHz, $CDCl_3$) δ: 7.33 – 7.22 (m, 2H), 7.06 – 6.93 (m, 2H), 6.92 – 6.78 (m, 1H), 3.67 (td, $J$ = 10.0, 4.5 Hz, 1H), 3.40 (ddd, $J$ = 11.6, 9.8, 3.5 Hz, 1H), 2.80 (s, 3H), 2.25 – 2.15 (m, 1H), 1.82 – 1.68 (m, 3H), 1.49 – 1.21 (m, 5H).

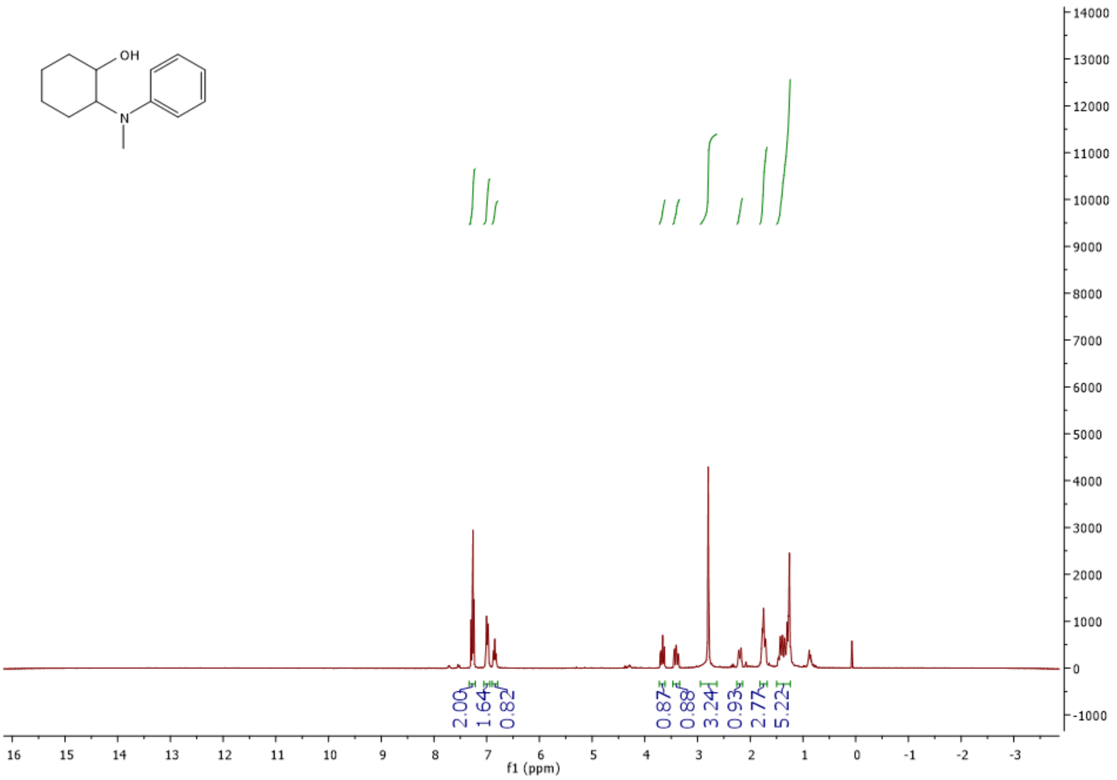

**2-(mesitylamino)cyclohexan-1-ol (16):** Following the general procedure described above, reaction of the epoxide **1** with amine **32** afforded the title compound **16** as an orange solid. Purification by flash column chromatography (hexane: ethyl acetate; 8:2). Yield: 75%. NMR data of **16** match with the previously reported in the literature. [13] $^1$H NMR (300 MHz, $CDCl_3$) δ: 6.83 (s, 2H), 3.47 (td, $J$ = 10.0, 4.5 Hz, 1H), 2.87 – 2.77 (m, 1H), 2.28 (s, 6H), 2.23 (s, 3H), 2.19 – 2.10 (m, 1H), 1.90 – 1.77 (m, 1H), 1.78 – 1.69 (m, 1H), 1.70 – 1.61 (m, 1H), 1.44 – 1.18 (m, 2H), 1.22 – 1.03 (m, 2H).

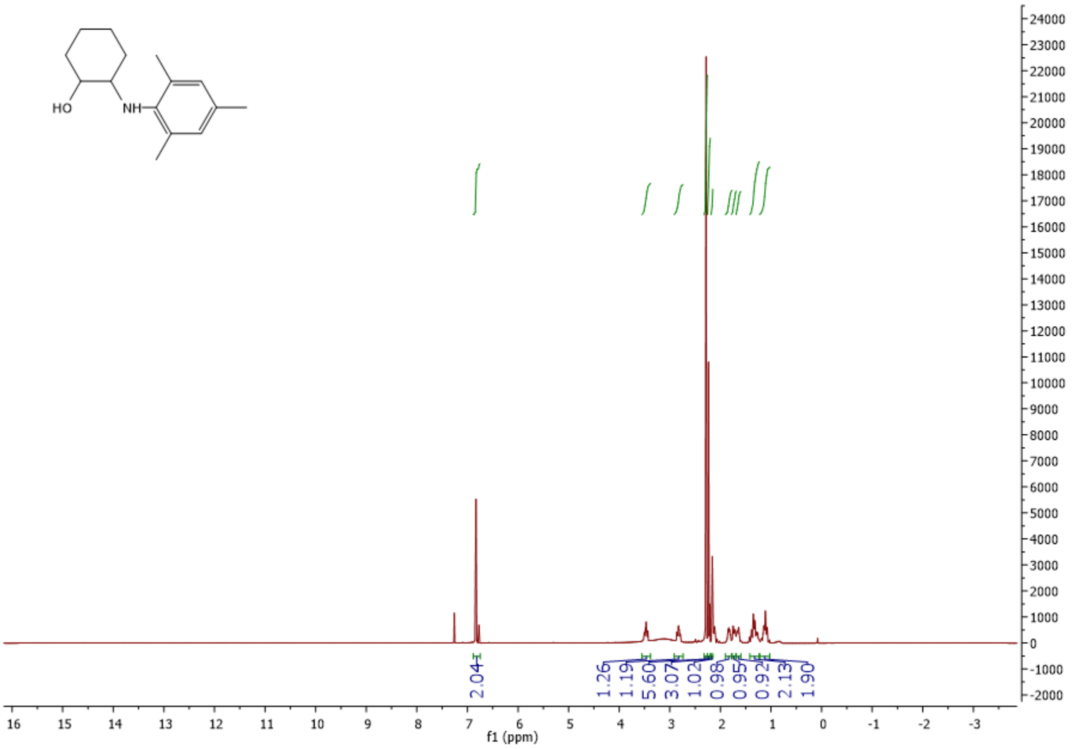

**1-(isopropylamino)-3-(naphthalen-1-yloxy)propan-2-ol (19):** reaction of the epoxide **17** with amine **18** afforded the title compound **19** as a brown solid . Purification by flash column chromatography (DCM: MeOH; 9:1). Yield: 92%. NMR data of **19** match with the previously reported in the literature. [21] $^{1}H$ NMR (300 MHz, $CDCl_3$) δ: 8.33 – 8.16 (m, 1H), 7.85 – 7.74 (m, 1H), 7.61 – 7.31 (m, 4H), 6.84 (dd, *J* = 7.5, 0.9 Hz, 1H), 4.28 – 4.06 (m, 3H), 3.10 – 2.93 (m, 1H), 2.94 – 2.78 (m, 2H), 1.11 (d, *J* = 6.3 Hz, 6H).

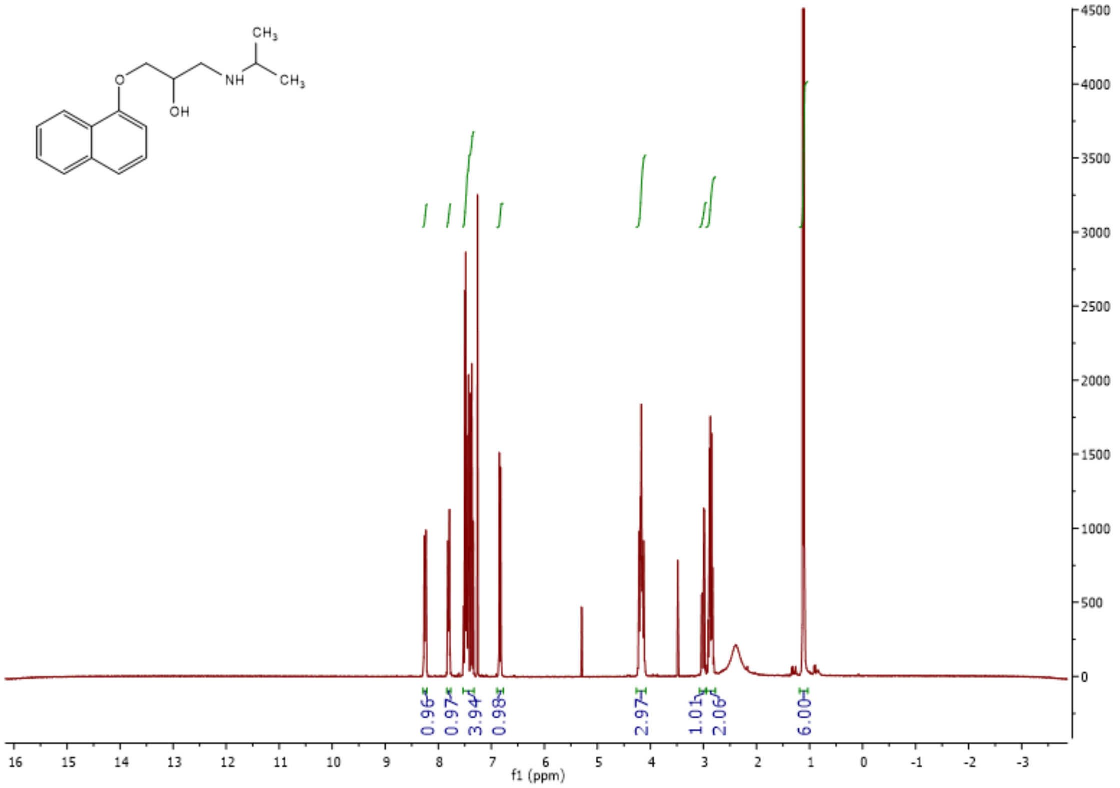

## S.9 REFERENCES

[1] Castells-Gil, J.; M. Padial, N. ; Almora-Barrios, N.; da Silva, I.; Mateo, D.; Albero, J.; García, H.; Martí-Gastaldo, C. De novo synthesis of mesoporous photoactive titanium(IV)–organic frameworks with MIL-100 topology. *Chem. Sci.* **2019**, *10*, 4313.

[2] Horcajada, P.; Surblé, S.; Serre, C.; Hong, D.-Y.; Seo, Y.-K.; Chang, J.-S.; Grenèche, J.-M.; Margiolaki, I.; Férey, G. Synthesis and catalytic properties of MIL-100(Fe), an iron (III) carboxylate with large pores. *Chem. Commun.* **2007**, 2820-2822

[3] Castells-Gil, J.; M. Padial, N. ; Almora-Barrios, N.; Gil-San-Millán, R.; Romero-Ángel, M.; Torres, V.; da Silva, I.; Vieira, B. C. J.; Waerenborgh, J. C.; Jagiello, J.; Navarro, J. A. R.; Tatay, S.; Martí-Gastaldo, C. Heterometallic Titanium-Organic Frameworks as Dual-Metal Catalysts for Synergistic Non-buffered Hydrolysis of Nerve Agent Simulants. *Chem* **2020**, *6*, 3118-3131.

[4] P. Atkins, J. de Paula in *Physical Chemistry*, Eight Edition, W. H. Freeman and Company, New York, **2006**, pp.798-800.

[5] Manjunathan, P.; Prasanna, V.; Shanbhag, G. V. Exploring tailor-made Brønsted acid sites in mesopores of tin oxide catalyst for β-alkoxy alcohol and amino alcohol syntheses. *Sci. Rep.* **2021**, *11*, 15718.

[6] Mizuno, K.; Ikeda, M.; Imokawa, T.; Take, J.-I.; Yoneda, Y. Nature of Catalytically Active Sites over Solid Acids. I. Selective Poisoning of Lewis Acid Sites on Silica-Alumina with Pyridine and Its Application to Olefin Polymerization. *Bull. Chem. Soc. Jpn.* **1976**, *49*, 1788-1793.

[7] Beyzavi, H.; Klet, R. C.; Tussupbayev, S.; Borycz, J.; Vermeulen, N. A.; Cramer, C. J.; Stoddart, J. F.; Hupp, J. T.; Farha, O. K. A Hafnium-Based Metal–Organic Framework as an Efficient and Multifunctional Catalyst for Facile CO2 Fixation and Regioselective and Enantioretentive Epoxide Activation. *J. Am. Chem. Soc.* **2014**, *136*, 15861-15864.

[8] Das, A.; Anbu, N.; Reinsch, H.; Dhakshinamoorthy, A.; Biswas, S. A Thiophene-2-carboxamide-Functionalized Zr(IV) Organic Framework as a Prolific and Recyclable Heterogeneous Catalyst for Regioselective Ring Opening of Epoxides. *Inorg. Chem.*, **2019**, *58*, 16581-16591.

[9] Tanaka, K.; Kinoshita, M.; Kayahara, J.; Uebayashi, Y.; Nakaji, K.; Morawiak, M.; Urbanczyk-Lipkowska, Z. Asymmetric ring-opening reaction of meso-epoxides with aromatic amines using homochiral metal–organic frameworks as recyclable heterogeneous catalysts. *RSC Adv.*, **2018**, *8*, 28139-28146.

[10] Tan, C.; Han, X.; Li, Z.; Liu, Y.; Cui, Y. Controlled Exchange of Achiral Linkers with Chiral Linkers in Zr-Based UiO-68 Metal–Organic Framework. *J. Am. Chem. Soc.*, **2018**, *140*, 16229-16236.

[11] Jiang, D.; Mallat, T.; Krumeich, F.; Baiker, A. Copper-based metal-organic framework for the facile ring-opening of epoxides. *J. Catal.* **2008**, *257*, 390-395.

[12] Pariyar, A.; Asl, J. Y.; Choudhury, A. Tetragonal versus Hexagonal: Structure-Dependent Catalytic Activity of Co/Zn Bimetallic Metal–Organic Frameworks. *Inorg. Chem.* **2016**, *55*, 9250-9257.

[13] Ji, P.; Feng, X.; Oliveres, P.; Li, Z.; Murakami, A.; Wang, C.; Lin, W. Strongly Lewis Acidic Metal–Organic Frameworks for Continuous Flow Catalysis. *J. Am. Chem. Soc.* **2019**, *141*, 14878-14888.

[14] Huang, Z.-Q.; Xu, Z.-H.; Liu, X.-H.; Zhao, Y.; Wang, P.; Liu, Z.-Q.; Sun, W.-Y. A novel copper framework with amino tridentate *N*-donor ligand as heterogeneous catalyst for ring opening of epoxides. *Appl. Organomet. Chem.* **2021**, *35*, e6262.

[15] Chakraborti, A. K.; Rudrawar, S.; Kondaskar, A. An efficient synthesis of 2-amino alcohols by silica gel catalysed opening of epoxide rings by amines. *Org. Biomol. Chem.* **2004**, *2*, 1277-1280.

[16] Mamillapalli, N. C.; Sekar, G. Chemoselective reduction of α-keto amides using nickel catalysts. *Chem. Commun.* **2014**, *50*, 7881-7884.

[17] Karimian, R.; Piri, F.; Karimi, B.; Abolghasem, M. Silica Chloride Nano Particle Catalyzed Ring Opening of Epoxides by Aromatic Amines. *Chin. J. Chem.* **2011**, *29*, 955-958.

[18] Chen, X.; Wu, H.; Wang, S.; Huang, S. Nano-$TiO_2$: An Efficient and Reusable Heterogeneous Catalyst for Ring Opening of Epoxides Under Solvent-Free Conditions. *Synth. Commun.* **2012**, *42*, 2440-2452.

[19] Curini, M.; Epifanio, F.; Marcotullio, M. C.; Rosati, O. Zirconium Sulfophenyl Phosphonate as a Heterogeneous Catalyst in the Preparation of β-Amino Alcohols from Epoxides. *Eur. J. Org. Chem* **2001**, 4149-4152.

[20] Shivani; Pujala, B.; Chakraborti, A, K. Zinc(**II**) Perchlorate Hexahydrate Catalyzed Opening of Epoxide Ring by Amines: Applications to Synthesis of (*RS*)/(*R*)-Propranolols and (*RS*)/(*R*)/(*S*)-Naftopidils. *J. Org. Chem.* **2007**, *72*, 3713-3722.

[21] Fagerström, A.; Nilsson, M.; Berg, U.; Isaksson, R. New propranolol analogues: binding and chiral discrimination by cellobiohydrolase Cel7A. *Org. Biomol. Chem* **2006***, 4*, 3067-3076.
